# Tonsillectomy versus tonsillotomy for recurrent acute tonsillitis in children and adults (TOTO): study protocol for a randomized non-inferiority trial

**DOI:** 10.1186/s13063-021-05434-y

**Published:** 2021-07-22

**Authors:** Orlando Guntinas-Lichius, Katharina Geißler, Thomas Asendorf, Ralf Tostmann, Jan Löhler

**Affiliations:** 1 Department of Otorhinolaryngology, Jena University Hospital, Am Klinikum 1, 07747 Jena, Germany; 2 The German Study Centre for Otorhinolaryngology, Head and Neck Surgery (DSZ-HNO), Bonn, Germany; 3 Department of Medical Statistics, University Medical Center Göttingen, Göttingen, Germany; 4 Study Center, University Medical Center Göttingen, Göttingen, Germany; 5 Scientific Institute for Applied ENT-Research (WIAHNO) of the German Professional Association of ENT-Surgeons (BVHNO), Bad Bramstedt, Germany

**Keywords:** Tonsillitis, Sore throat, Tonsillectomy, Tonsillotomy, Surgery, Randomized controlled trial

## Abstract

**Background:**

Tonsillectomy is one of the most frequently performed surgeries in children and young adults worldwide. For decades, tonsillectomy was the surgical treatment of choice for recurrent acute tonsillitis. Tonsillotomy was used in some countries as an alternative to tonsillectomy only for the treatment of obstructive sleep apnea in young children. In recent years, an increase of tonsillotomy also to treat recurrent acute tonsillitis can be observed. Therefore, the German Institute for Quality and Efficiency in Health Care (IQWiG) was commissioned by the Federal Joint Committee (G-BA) to investigate whether tonsillotomy offers advantages compared to tonsillectomy. The meta-analysis of the IQWiG including studies until 2016 revealed that the long-term benefits and harms of tonsillotomy compared to tonsillectomy are unclear. Consequently, the G-BA performed a European call for a clinical trial. A consortium of the German Professional Association of ENT-surgeons (BVHNO), the German Society of Oto-Rhino-Laryngology, Head and Neck Surgery (DGHNO-KHC), and the Jena University Hospital were finally selected to perform the TOTO study.

**Methods:**

TOTO is a multicenter, 1:1 two-arm, randomized non-blinded non-inferiority trial. Four hundred fifty-four patients ≥ 3 years of age will be randomly allocated to undergo either tonsillotomy or tonsillectomy as surgical treatment of recurrent acute tonsillitis. All participants will be followed up for a total of 24 months. The primary outcome is the number of sore throat days experienced over the 24-month follow-up.

**Discussion:**

TOTO is designed to evaluate the effectiveness and efficiency of tonsillectomy versus tonsillectomy for the management of patients with recurrent acute tonsillitis. Tonsil disease and surgery have a major impact on preschool and school children as well as on economically active young adults, with individual and societal costs through loss of school visits, earnings, and productivity. If tonsillotomy is at least as effective as tonsillectomy but with reduced morbidity, this would reduce costs to the healthcare system and society.

**Trial registration:**

German Clinical Trials Register DRKS00020823. Registered on 04 September 2020.

**Supplementary Information:**

The online version contains supplementary material available at 10.1186/s13063-021-05434-y.

## Administrative information

The order of the items has been modified to group similar items (see http://www.equator-network.org/reporting-guidelines/spirit-2013-statement-defining-standard-protocol-items-for-clinical-trials/).

**Table:** 

Title {1}	Tonsillectomy versus tonsillotomy for recurrent acute tonsillitis in children and adults (TOTO): a randomized open-label multi-center non-inferiority clinical trial
Trial registration {2a and 2b}.	German Clinical Trials Register, DRKS00020823, date of registration: 04-September-2020
Protocol version {3}	V 1.1, 08-May-2020
Funding {4}	Study sponsored by the Federal Joint Committee (Gemeinsamer Bundesausschuss; G-BA)
Author details {5a}	Orlando Guntinas-Lichius^1,2^, Katharina Geißler^1^, Thomas Asendorf^3^, Ralf Tostmann^2,4^, Jan Löhler^2,5^ ^1^Department of Otorhinolaryngology, Jena University Hospital, Jena, Germany ^2^The German Study Centre for Otorhinolaryngology, Head and Neck Surgery (DSZ-HNO), Bonn, Germany ^3^Department of Medical Statistics, University Medical Center Göttingen, Göttingen, Germany ^4^Study Center, University Medical Center Göttingen, Göttingen, Germany ^5^Scientific Institute for Applied ENT-Research (WIAHNO) of the German Professional Association of ENT-Surgeons (BVHNO), Bad Bramstedt, Germany
Name and contact information for the trial sponsor {5b}	Jena University Hospital, Am Klinikum 1, 07747 Jena, Germany
Role of sponsor {5c}	Study protocol development and study funded by the Federal Joint Committee (Gemeinsamer Bundesausschuss; G-BA). The choice of the comparators (tonsillotomy versus tonsillectomy) was predetermined by the G-BA. The study sponsor did not influence study design and will not influence the collection, management, analysis, and interpretation of data; writing of the report; and the decision to submit the report for publication. The sponsor will not have any authority over any of these activities.

## Introduction

### Background and rationale {6a}

Tonsil surgery still is one of the most frequent otolaryngological surgeries, especially in children and young adults. Although the annual surgical rates have continuously declined since 2005, in 2018, still 77 tonsillectomies per 100,000 habitants and 25 adenotonsillectomies were performed [1]. At least since the publication of the Bertelsmann Foundation report “Health fact check: removal of tonsils in children and adolescents” in 2013, a new discussion about clear indication criteria for tonsillectomy was stimulated [2]. This report observed a very high regional variance in the number of tonsil surgeries. The authors could not find any medical reason justifying this variance. In parallel, the Austrian Societies of Otorhinolaryngology, Head and Neck Surgery and Paediatrics published in 2013 the results of a nationwide multicenter study on tonsil surgery based on the release of a consensus paper with clear recommendations for tonsil surgery from 2007 [3]. The aim was to restrict tonsillectomies and to promote tonsillotomy as an alternative because a series of deaths after severe post-tonsillectomy hemorrhage in children were observed in Austria in 2006 and 2007. Subsequently, a decrease of tonsillectomies could be observed and lower morbidity after tonsillotomy. In Germany, this aroused the desire for better quality assurance procedures for the indication of a tonsillectomy or a tonsillotomy. An important subsequent step was the introduction of the clinical guideline “Therapy of inflammatory conditions of the tonsils - tonsillitis” under the leadership of the German Society for Ear, Nose, and Throat, Head and Neck Surgery in 2015 [4]. This guideline defined clear recommendations for an indication for tonsil surgery in case of recurrent acute tonsillitis. A potential indication for tonsillectomy or tonsillotomy is the same in this guideline: ≥ 6 episodes of physician-diagnosed tonsillitis treated with antibiotics within the last 12 months or 3–5 episodes, if further episodes occur in the next 6 months and a total number of 6 episodes is reached. The introduction of the guideline was followed in Germany by a vigorous debate because of the strict criteria and the quasi-equation of both surgical methods. Also in 2015, the recruitment of the NAtional Trial of Tonsillectomy IN Adults (NATTINA) started in the UK [5]. NATTINA has another important focus. NATTINA is a multicenter, randomized, controlled trial for adults with recurrent tonsillitis to compare the clinical and cost-effectiveness of tonsillectomy versus conservative management. NATTINA is meanwhile closed and the results are awaited.

In 2016, the independent German Institute for Quality and Efficiency in Health Care (Institut für Qualität und Wirtschaftlichkeit im Gesundheitswesen, IQWiG) was asked by the Federal Joint Committee (G-BA) to examine whether tonsillotomy offers advantages over tonsillectomy in case of tonsil hyperplasia and obstructive sleep disorders as well as for recurrent acute tonsillitis. The resulting IQWiG report N15-11 was published in 2017 [6]. The IQWiG finally identified 19 relevant randomized controlled trials including 1590 patients. Essentially, the IQWiG report identified short-term benefits for tonsillotomy, but it was said that long-term benefits and harms are unclear. A systematic review (without meta-analysis) from 2018 screened 512 articles until April 2017 and included nine trials reporting on 770 patients [7]. The authors of this recent review concluded that the data suggest an equal efficacy of tonsillotomy and tonsillectomy in adults and a preference for tonsillotomy in terms of pain, analgesics use, patient-satisfaction, operation time, and postoperative complications.

Because of the IQWiG report, the G-BA included in 2019 tonsillotomy in the health insurance catalog for outpatient surgery for the treatment of palatine tonsil hyperplasia. On the other hand, the G-BA stated that the benefit for the treatment of recurrent acute tonsillitis is unclear and that a clinical trial is needed to provide information on long-term benefits and risks of tonsillotomy to treat recurrent acute tonsillitis. The G-BA conducted a European-wide tender for this trial. The TOTO consortium won this tender end of 2019 and was given 6 months to develop a study protocol. This protocol is presented here.

### Objectives {7}

The primary study objective is to compare the frequency of sore throat episodes within 24 months after tonsillotomy versus tonsillectomy in children and adults treated for recurrent acute tonsillitis. We hypothesize that the frequency of sore throat episodes is not higher after tonsillotomy compared to tonsillectomy. Secondary objectives are the frequency of sore throat episodes within the first 12 months after surgery, the days of incapacity of work/out of school, tonsil-specific quality of life, need for treatment with antibiotics, and need for treatment with analgesics within 24 months after surgery. Furthermore, mortality and morbidity (primary and secondary bleedings and other intraoperative and postoperative complications will be evaluated as well as the need for re-surgery within 24 months.

### Trial design {8}

**Fig. 1:**
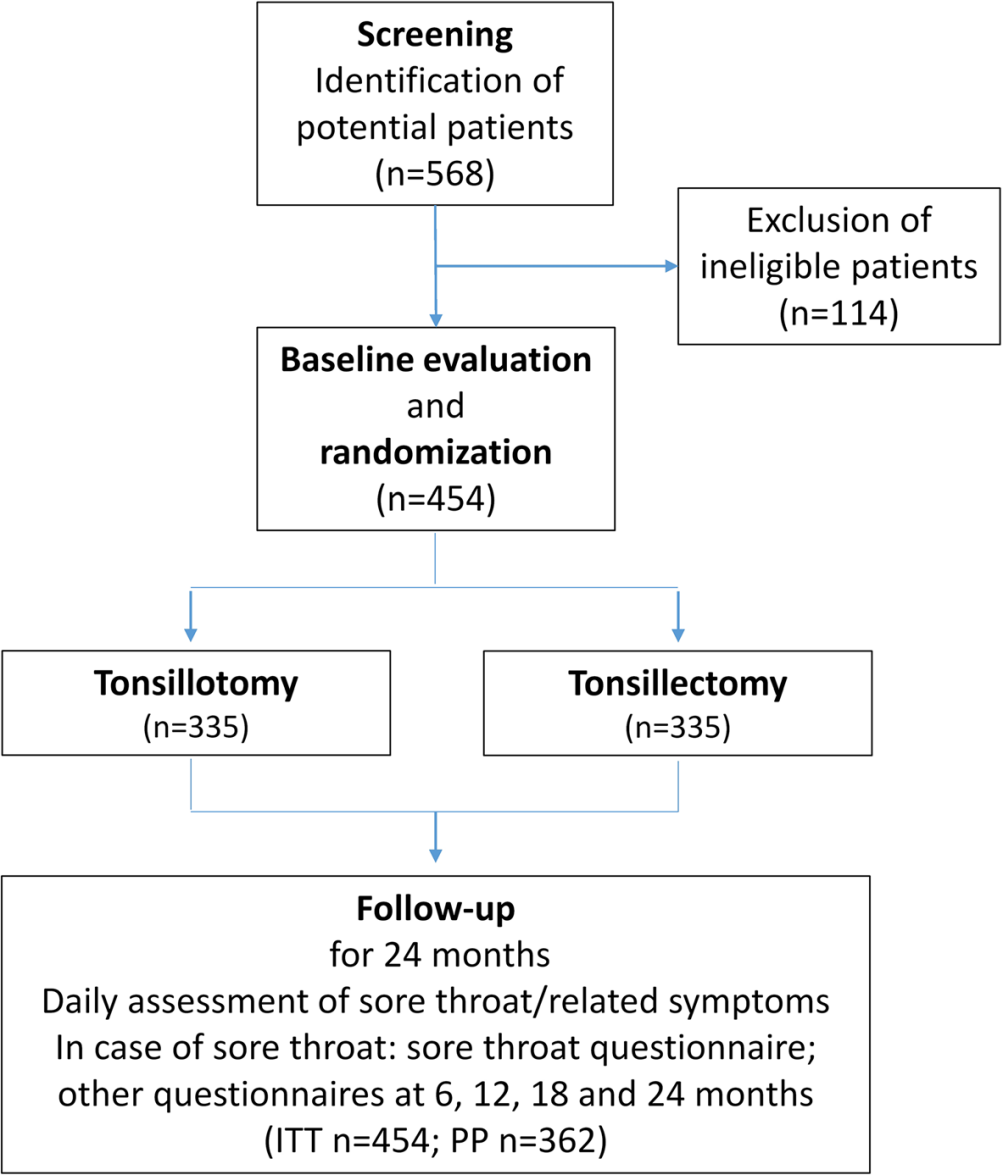
Flow chart of the trial process from allocation to follow-up. ITT, intention to treat; PP, per protocol

This study is a multicenter, two-arm (parallel group), randomized (1:1) open-label non-inferiority trial. Patients will be randomly allocated to undergo either tonsillotomy or tonsillectomy as surgical treatment of recurrent acute tonsillitis. Additional adenoidectomy is allowed in both arms. A flowchart of the study is presented in Fig. 1.

## Methods: Participants, interventions, and outcomes

### Study setting {9}

Patients will be recruited from twenty hospitals (in-patient and out-patient) across Germany. An up-to-date list of study sites will be published online on the TOTO homepage (https://toto-studie.hno.org/). Only study centers with at least 10 tonsil surgeries and a principal investigator with GCP certificate are eligible.

### Eligibility criteria {10}

#### Inclusion criteria

- Age ≥ 3 years
- ≥ 6 episodes of physician-diagnosed tonsillitis treated with antibiotics within the last 12 months or 3–5 episodes, if (a) further episodes occur in the next 6 months and a total number of 6 episodes is reached, or (b) there are additional patient-specific factors (special personal or professional concern, severe symptoms during the tonsillitis episodes, individual patient request) according to the German guideline for the treatment of tonsillitis [4].
- Subject (or parents/caregivers in case of underage participants) has provided written informed consent for participation in the study prior to any study-specific procedures

#### Exclusion criteria

- Under 3 years of age
- Previous tonsillotomy or tonsillectomy
- Other diseases of the tonsils including non-inflammatory tonsillar hyperplasia, peritonsillar abscess/quinsy, primary sleep breathing disorder, suspected malignancy, pregnant or breastfeeding, bleeding diathesis, therapeutic anticoagulation, craniofacial dysmorphism.
- Tonsillitis due to other reasons including mononucleosis, or insufficient knowledge of the German language to understand the questionnaires.

### Who will take informed consent? {26a}

The clinical team at the participating sites will approach and screen all children and adults who will undergo an elective (adeno)tonsillotomy or (adeno)tonsillectomy for the inclusion criteria. Potential participants will be shown the study information at their first clinic visit (unless already viewed online) and allowed to discuss the study with the designated member of the research team. Inclusion and exclusion criteria will be checked and eligible patients invited to participate in the study. Participants will be given at a minimum of 24 h to decide whether they would like to participate. Eligible patients/parents wishing to take part will be asked to provide written informed consent by signing and dating the Informed Consent Form (ICF), which will be witnessed, signed, and dated by a member of the research team. The original signed consent form will be retained in the Investigator Site File, with a copy in the clinical notes and a copy provided to the participant/parents. The study teams at each site will receive standardized, study-specific education based on standard study education materials provided by the central study administration. Notification letters about the study will be sent to relevant physicians with consultation or referral roles at study sites.

### Additional consent provisions for collection and use of participant data and biological specimens {26b}

Not applicable as no biological specimens are collected as part of this trial.

## Interventions

### Explanation for the choice of comparators {6b}

The choice of the comparators (tonsillotomy versus tonsillectomy) was predetermined by the European tender of the G-BA. The G-BA has tendered this study because the preceding assessment could not clarify if a tonsillotomy and tonsillectomy are equally efficacious in patients with recurrent acute tonsillitis and indication for tonsil surgery.

### Intervention description {11a}

Patients in the treatment arm will undergo a tonsillotomy. Tonsillotomy is defined as bilateral partial resection of the palatine tonsils. The resection is performed in such a way, that the remaining parts of the tonsil do not protrude the level of the anterior and posterior palatal arch in a non-luxated situation. Patients in the control arm will undergo tonsillectomy. Tonsillectomy is defined as a complete extracapsular resection of both palatine tonsils. An additional adenoidectomy is allowed. The following surgical techniques are allowed: cold instruments (i.e. scissors), CO_2_-laser, coblation, argon plasma coagulation, or radiofrequency ablation.

### Criteria for discontinuing or modifying allocated interventions {11b}

If the assigned type of tonsil surgery is discontinued or modified, the patient has to be excluded from the per-protocol analysis.

### Strategies to improve adherence to interventions {11c}

The participants have no influence on the adherence to both surgical interventions. Both surgeries are standard procedures for the ENT surgeons in the study centers. A variability of tonsillectomy can be excluded. To control the resection extent in tonsillotomy, the ENT surgeons in the study centers will be instructed to adhere to the resection in the level of the palatine arches.

### Relevant concomitant care permitted or prohibited during the trial {11d}

There are no relevant concomitant care and interventions that are permitted or prohibited during the trial. The use of analgesics and antibiotics is a secondary outcome measure of the trial.

### Provisions for post-trial care {30}

Not applicable as no provisions for post-trail care are part of this trial.

### Outcomes {12}

#### Primary outcome measure

- The number of symptomatic sore throat episodes within 24 months after surgery, aggregated through reports of daily sore throat activity (collected once per week as patient-reported outcomes)
- Symptomatic sore throat episodes occur by definition when a sore throat has been reported for at least three consecutive days. Two measurements of sore throat that are at least four days apart are considered different symptomatic sore throat episodes [8]

#### Secondary outcomes measures

- The number of symptomatic sore throat episodes within the first 12 months
- The number of days of incapacity for work/inability to go to the kindergarten/school and sore throat related limitation in daily routine cumulated at 6, 12, 18, and 24 months
- Tonsil and Adenoid Health Status Instrument (TAHSI) and the Tonsillitis Outcome Inventory-14 (TOI-14) prior and after surgery at 6, 12, 18, and 24 months [9–11]
- Quality of life assessed by Short Form-12 (SF-12) Questionnaire prior to surgery and after surgery at 6, 12, 18, and 24 months [12]
- Cumulative number of days using analgesics because of sore throat within 24 months
- Cumulative number of days using antibiotics because of sore throat within 24 months
- Safety analyses: morbidity and mortality after surgery, primary/secondary bleedings within 24 months; intraoperative and postoperative complications
- Revision tonsil surgery within 24 months

### Participant timeline {13}

Table 1 gives an overview of the participant timeline. Baseline characteristics of the subjects will be compiled at informed consent. Subjects will be randomly divided into an intervention arm and a control arm with the aid of a computer-based randomization module in a 1:1 ratio. Stratified randomization is accomplished using age group (< 18 years, ≥ 18 years and study center. All tonsillotomies and tonsillectomies will be carried out under general anesthesia. Surgery will be performed by all grades of doctors, from residents to specialists. Which from the allowed techniques is chosen depends on the surgeon’s experience and preference. If a tonsillotomy was performed, the study center has to decide, if the patient is treated as a day care case or if a therapy as an in-patient is indicated. All tonsillectomy patients are treated as in-patients. After surgery, all patients will be kept in the ward for at least 3–5 days. All patients receive standard pain management depending on the study center choice. After discharge, the participants will primarily be contacted by a mobile application called the TOTO app designed to run on mobile devices like mobile phones, tablet, or watch. Alternatively, the participant can enter the data via a link on the internet. Participants/parents who do not have a needed electronic device will receive a paper-based diary. We assume that about 75% of the participants are able to use the app. Using the app, the participants will be contacted weekly with a push-function of the app and asked if they had sore throat in the previous 7 days. If the participant did not experience sore throat in the last 7 days, the questioning ends. If the participant experienced sore throat in the last 7 days, the app automatically opens a sore throat questionnaire with ten questions on pain intensity and sore throat-related limitations. Furthermore, each study center will contact their participants for a telephone interview after 6, 12, 18, and 24 months. At these four times points, the participants should answer again the TAHSI, the TOI-14, and the SF-12 questionnaire.

**Table 1:** Standard Protocol Items of the TOTO trial: Recommendations for Interventional Trials (SPIRIT) table of the trial schedule for enrolment, interventions, and follow-up/assessments

			Study period							
	Clinic visit	Admission	Post-allocation							Close-out
Timepoints	Registry	Scheduled	0	POD1-5	Weekly to POM 24	POM 6	POM 12	POM 18	POM 24	POM 24
**Enrollment**										
**Eligibility screen**	X									
**Informed consent**	X									
**Baseline information**	X									
**Allocation**		X								
**Interventions**										
**Tonsillotomy**			X							
**Tonsillectomy**			X							
**Assessments**										
**Demographic data**	X	X								
**Additional Adenoidectomy**		X								
**TAHSI**	X					X	X	X	X	
**TOI-14**	X					X	X	X	X	
**SF-12**	X					X	X	X	X	
**Weekly App alert**					X					
**If sore throat: sore throat questionnaire**					X					
**Complications, AE**			X	X	X					X
**Participation sign-off**										X

*AE* adverse event, *NRS* numeric rating scale, *POD* postoperative day, *POM* postoperative month, *SF* Short Form Health Survey, *TAHSI* Tonsil and Adenoid Health Status Instrument, *TOI* Tonsillectomy Outcome Inventory

### Sample size {14}

Non-inferiority of a tonsillotomy compared to tonsillectomy is given when the threshold of the lower limit of the 95% confidence interval for a difference in the number of days of sore throat is above 80%. Assuming a rate of 7.56 days within 24 months in both groups, a dispersion parameter of 0.44, and a significance level of 2.5%, a negative binomial regression analysis would detect a significant difference of the quotient of the rates compared to a non-inferiority limit of 0.8 with an 80% power, if 181 participants were treated in both arms. Assuming a loss of follow-up data of 20%, 454 patients have to be randomized.

### Recruitment {15}

To achieve adequate participant enrollment, we contacted beforehand eligible study centers with a minimum of 10 tonsil surgeries per year beforehand. Based on the letters of intent of the first 20 responses, the target numbers for 20 study centers reached already 947 cases for a recruitment period of 24 months. Therefore, we plan 20 study centers for TOTO. For the unlikely event of insufficient recruitment, we have about 10 other study centers on a waiting list.

## Assignment of interventions: allocation

### Sequence generation {16a}, concealment mechanism {16b}, and implementation {16c}

Block randomization with random block lengths will be used to allocate participants to the two groups of tonsil surgery in a 1:1 ratio, stratified by center and age (< 18 years, ≥ 18 years). Randomization will be administered centrally via the TOTO organization team using a web-based system, accessible by the principal investigator or delegated individual of each study center. The randomization list will be computer generated and provided by the trial statistician. On-site study personnel will perform the randomization.

## Assignment of interventions: blinding

### Who will be blinded {17a}

Participants and involved otolaryngologists will not be blinded. Blinding is not possible because it will be obvious for the participant/parents and involved otolaryngologists when looking into the mouth, if a tonsillotomy or a tonsillectomy was performed. Furthermore, postoperative pain is normally much lower after tonsillotomy. The involved biostatistician will be blinded until database lock. The assessment of the primary endpoint and secondary endpoints is following a strict evaluation algorithm. The blinded statistician and an independent otolaryngologist make up the endpoint committee. If an individual evaluation of an endpoint remains unclear, the endpoint committee will discuss the case. All confirmatory analyses will be comprehensively specified in the predefined statistical analysis plan to avoid any reporting bias.

### Procedure for unblinding if needed {17b}

Not applicable as no unblinding is part of this trial.

## Data collection and management

### Plans for assessment and collection of outcomes {18a}

All participants will be followed up for 24 months from the date of randomization. Once consented, the baseline data collection of the participant will take place in the study center. The preoperative patient-reported outcome measures (PROMs) will be performed. The PROMs include the internationally used and validated general quality of life Short Form-12 (SF-12) questionnaire [12]. Furthermore, the Tonsil and Adenoid Health Status Instrument (TAHSI) questionnaire and the Tonsillectomy Outcome Inventory 14 (TOI-14) will be used [10, 11, 13]. The German version of the TAHSI is only validated for adults, although it has been used already also for German children. However, because no other comparable PROM is available, we decided to use the German version of the TAHSI for adults and children to use a uniform study design and ensure comparability [14]. The TOI-14 was primarily developed and validated in German for adults with chronic tonsillitis (10). After discharge, the further collection of outcomes is performed at home. The participants/parents will be asked weekly via the TOTO app for sore throat during the previous 7 days. The participants can answer directly in the app or using a link via a web browser. If the participant had no sore throat, the request is finished. In case of sore throat, ten questions are asked to provide details of the severity, use of analgesics and antibiotics, and number of days when unable to undertake usual activities (including work/school/studies) are taken. Participants not using the app, use weekly documentations in the TOTO diary. In addition, the study center will call the participants at 6, 12, 18, and 28 months after inclusion. The 6-monthly telephone interviews will include the same questionnaires as preoperatively, i.e. TASHI, TOI-14, and SF12.

### Plans to promote participant retention and complete follow-up {18b}

The primary objective of the trial is to collect long-term data up to 24 months after tonsil surgery. The measurement of the days with sore throat and also the PROM results are depending on the information given by the participants. Therefore, a follow-up visit to the study center is not necessary. Furthermore, it can be presumed that the motivation of participants without any further complaints will decrease the longer the study takes. For these reasons, we decided to use a mobile application, i.e., the TOTO app, to collect the sore throat data during the follow-up. Using a push function, it is easy to prompt the participants and to remember the participants to answer the weekly questions. If the participant has no sore throat, the weekly push call via the app is answered with one click. The survey is only opened if the participant had a sore throat in the previous 7 days. Furthermore, the study center will call participants, if they have not answered through the app repeatedly. The diaries will be collected every 6 months.

### Data management {19}

For the pseudonymized collection of pseudonymized patient data, the coordinating center configures and provides an electronic case report form (eCRF) in good clinical practice (GCP)-compliant browser-based electronic data capture (EDC; SecuTrial®, Berlin, Germany). In particular, user rights can be managed via the EDC, data can be signed, any changes can be tracked via a prescribed audit trial, and the query management can be mapped. Data are entered at appropriate intervals after the source data has been created. Data are entered exclusively by authorized personnel at the study centers or, in the case of the PROMs, by the participants themselves using the TOTO app. The data are viewed on the principle of economy, i.e., each authorized user in the database can only view the data that is necessary for the fulfillment of his role. After completing the data entry and cleaning up the database, the data are handed over to the responsible biometrician for evaluation and a copy of the data for archiving to the sponsor.

The following source data are transferred from the center to the eCRF: inclusion criteria, exclusion criteria, demographic data (age, gender, employment relationship/child care relationship), additional adenoidectomy, TAHSI, TOI-14, SF-12, app usage, diary usage, documentation of adverse events (AEs). Each study center will be authorized to open a participant access to the database. The log-in takes place via the pseudonym assigned by the study center to ensure the anonymity of the participant data. The database access enables participants to make entries in the database either via the TOTO app or via a web-browser on the internet. The participants regularly provide the following data: day of sore throat (weekly basis), and in case of a sore throat, data on sore throat-related limitations, and drug use. The participant data are regularly checked for completeness. If the patient-reported outcomes are repeatedly not entered, this is automatically reported to the study center, which contacts the participant by phone to offer assistance. In addition, the reason for a possible non-entry is surveyed every 6 months for the telephone interview. If a paper-based diary is provided for participants who did not want to participate in electronic data collection, this diary is transferred from the study centers to the database every 6 months.

Several quality assurance measures will take effect at various levels to meet the quality of the data collected but also the requirements of the ICH GCP (as a reference system). At the implementation level, quality assurance will be carried out in accordance with the existing SOPs of the study center in Göttingen, Germany. This includes, among other things, that pre-study visits and initiation visits take place before the centers open and that the monitoring would be implemented according to a study-specific monitoring plan. As part of a risk-based management approach, risk-adapted monitoring and project management strategies will come into play. The data will be collected via a separate eCRF system (SecuTrial). This system uses a specific role concept with different authorizations for centers, monitors, and data managers. All data entries including deletions and changes can be tracked via an audit trail. Automatic checks for plausibility and correctness of the data will be implemented. Query management for questionable data entries is performed. The database is validated before commissioning. The database is not closed until the database is cleaned up.

### Confidentiality {27}

Personal data will be regarded as strictly confidential. A unique participant ID will be assigned to each participant at randomization. Participants/parents have to give consent for their contact details (name, address, phone numbers, and email address) to be used for the telephone interviews during follow-up. All electronic personal information will be kept on password-protected databases with restricted access and paper forms securely filed in a locked cabinet. Only the clinical team at the participating study center will have access to key data, which links study identifiers to individual datasets. All data transfer from the study centers and transferred from the participants directly via the app will be pseudonymized. These data are saved on local servers (RAID 10 system with daily backups) at the TOTO coordinating center Saved backups are available for up to 20 days. All processes comply with the requirements of the German general data protection regulations. All study records and Investigator Site Files will be kept GCP-conform at a site in a locked filing cabinet with restricted access.

Furthermore, a study-specific data protection concept will be developed. The patient-identifying information is only kept at the patient-treating center (the study center) and is stored with restricted access. Inspections are only carried out by specially authorized personnel (e.g., monitors) to whom reference was made in the declaration of consent. The anonymized data set will finally be published together with the primary results to ensure transparency.

### Plans for collection, laboratory evaluation, and storage of biological specimens for genetic or molecular analysis in this trial/future use {33}

Not applicable as no genetic or molecular analyses are part of this trial.

## Statistical methods

### Statistical methods for primary and secondary outcomes {20a}

Continuous variables will be presented as means and standard deviation or median and interquartile range (IQR) if not normally distributed. Categorical data will be presented as a number with a percentage. The primary analysis is performed on the per-protocol (PP) set and sensitivity analyses in the intention-to-treat (ITT) and full analysis population are also planned.

Differences between the observed risks of events (for primary outcomes of sore throat days) between both groups will be calculated along with their 95% confidence intervals by negative binomial regression. The non-inferiority of the tonsillotomy compared to the tonsillectomy in the number of symptomatic sore throat days within 24 months is evaluated by means of negative binomial regression with the stratification factors as a covariate and the non-inferiority margin of 0.8 to a one-sided significance level of 2.5%. In secondary outcome, the number of symptomatic sore throat days within 12 months after surgery is evaluated using the negative binomial regression with the stratification factors as a covariate and the non-inferiority margin of 0.8. Other parameters like the PROM results are evaluated using analyses of covariance (ANCOVA), with the stratification variables and baseline observations as covariates. Number of days with the inability to work/attend kindergarten/school after 24 months are evaluated by negative binomial regression with stratification variables as covariates. A one-sided significance level of 2.5% will be considered statistically significant (or 5% when testing two-sided). All confirmatory analyses are fully specified in a statistical analysis plan (SAP), which is written before database lock and before unblinding the trial statistician, in order to avoid reporting bias. The results are published independently of the statistical results in order to avoid publication bias.

### Interim analyses {21b}

An interim analysis is not planned.

### Methods for additional analyses (e.g., subgroup analyses) {20b}

Subgroup analysis is planned for the subgroups of working participants and kindergarten/school visitors. The number of days with the inability to work or inability to attend kindergarten/school at the times 6 months, 12 months, 18 months, and 24 months are evaluated descriptively using the negative binomial regression in these subgroups

### Methods in analysis to handle protocol non-adherence and any statistical methods to handle missing data {20c}

The primary hypothesis of non-inferiority is carried out in the per protocol (PP) population. The criteria for serious protocol deviations that lead to exclusion from the analysis are recorded in SAP before the end of the data collection. The intention-to-treat population (ITT) contains all patients who were randomized to one of the treatments and also assigns them to the randomized treatment group. The non-inferiority is only tested in this population as a sensitivity analysis. Missing values are replaced with the help of multiple imputations. The imputation method is described in detail in SAP. The full analysis population consists of all participants who were randomized and operated on and who provided at least one indication of the occurrence of a sore throat. Missing values are also replaced with the help of multiple imputations. Finally, the safety analysis population consists of all patients who underwent surgery.

### Plans to give access to the full protocol, participant level-data and statistical code {31c}

No later than 3 years after the end of the trial, we plan to deliver a completely de-identified data set to an appropriate data archive for sharing purposes.

## Oversight and monitoring

### Composition of the coordinating center and trial steering committee {5d}

The coordinating study center is the Department of Otolaryngology, Jena University Hospital. The trial steering committee (TSC) consists of members of the Jena University Hospital, Germany, of the clinical trial center, University Medical Center, Göttingen, Germany, and the lead investigators in each participating study center. The Trial Management Committee (TMC) consists of the chief investigator, members of the BVHNO, the DGHNO-KHC, and of the clinical trial center, University Medical Center, Göttingen, Germany. The TMC was responsible for all aspects of the study planning, and will be responsible for the randomization, data registration, pharmacovigilance, data management, biostatistics and study monitoring. The TMC also organizes the TSC meetings.

### Composition of the data monitoring committee, its role and reporting structure {21a}

An independent data monitoring committee (DMC) is established. The DMC can make recommendations independent of the sponsor, the coordinating study center, or of the TSC. The function of the DMC is to check predefined data sets and data points in a defined frequency and to assess whether, on the one hand, study participant safety is still guaranteed and whether, on the other hand, the conduct of the study is still ethically responsible and whether the fundamental benefit assessments have shown changes. The TOTO DMC charter will describe the composition, the tasks, the organizational structure, the review, and decision-making processes as well as the reporting to and by the DMC in detail. The DMC charter will be available on request from the TSC.

### Adverse event reporting and harms {22}

An adverse event (AE) will be defined as any untoward medical occurrence in a participant without regard to the possibility of a causal relationship. AEs will be collected after the participant has provided consent and enrolled in the study. If a participant experiences an AE after the informed consent document is signed (entry) but the participant has not started to receive study intervention, the event will be reported as not related to the intervention. All AEs occurring after the entry into the study and until discharge from the study will be recorded. After discharge from the hospital, i.e., during the follow-up phase at home, the participants are asked to report AE at least during the weekly app consultation. An AE that meets the criteria for a serious adverse event (SAE) between study enrollment and discharge will be reported to the local Ethics committee and the sponsor as an SAE. If the tonsil surgery is discontinued as a result of an AE, study personnel will document the circumstances and data leading to the discontinuation of treatment. A SAE for this study is any untoward medical occurrence that is believed by the investigators to be causally related to study intervention and results in any of the following: life-threatening condition (that is, immediate risk of death); severe or permanent disability, prolonged hospitalization, or a significant hazard as determined by the DMC. SAE occurring after a participant is discontinued from the study will NOT be reported unless the investigators feel that the event may have been caused by the study drug or a protocol procedure. Investigators will determine the relatedness of an event to study intervention based on a temporal relationship to the study intervention, as well as whether the event is unexpected or unexplained given the subject’s clinical course, previous medical conditions, and concomitant medications.

### Frequency and plans for auditing trial conduct {23}

Monitoring and source data verification are the important parts f the auditing trial conduct independent from investigators and the sponsor.

#### Monitoring

The investigator in each study center grants the monitor access to the participant’s medical records to verify the proper documentation of the study data. The provisions of the Federal Data Protection Act are fully respected. The monitor is bound to the contractual confidentiality regulations when comparing the CRFs with the source documents. All investigators agree that the monitors can visit the study center before, during, and after the study is completed. The investigator must allow enough time for these visits. Alternatively, other trained personnel (e.g., so-called co-examiners) can be made available to the monitor for support during the visits. The reviewer grants the monitor access to the source documents to perform its duty. The aim and purpose of these visits are in particular: assessment of the progress of the study, checking compliance with the study protocol, CRF check for correctness and completeness, and CRF validation and source data comparison. A monitor report is created for each visit. This documents the progress of the study and provides a report on all problems that have occurred (e.g., deviations from the study protocol. The monitor will also hold a contact to the centers by phone between visits.

#### Source data verification (SDV)

The monitoring is also important for the source data check. The source data is checked in order to check the correctness and completeness of the entries in the CRF by comparison with the original data and thus to ensure and improve the quality of the data. All data required for SDV must have been entered in the medical record or, for source documents, in the medical record. The investigators grant the monitor access to the medical documents for performing the source data comparison. The source data defined by ICH-GCP include data such as hospital records, clinical diagrams, patient diaries or evaluation checklists, copies or transcriptions that have been certified as such after verification, exact copies, microfiches, photographic negatives, microfilms or magnetic media, telephone surveys can - after signing off by the examiner - considered as source data.

### Plans for communicating important protocol amendments to relevant parties (e.g., trial participants, ethical committees) {25}

Any modifications to the protocol which may impact on the conduct of the study, a potential benefit of the patient or may affect patient safety, including changes of study objectives, study design, patient population, sample sizes, study procedures, or significant administrative aspects will require a formal amendment to the protocol. Such an amendment will be agreed upon by TSC and the sponsor and approved by the Ethics Committee prior to implementation. The amendments will be notified to the health authorities in accordance with local regulations. Administrative changes of the protocol are minor corrections and/or clarifications that do not affect on the way the study is to be conducted. These administrative changes will be agreed upon by the TSC and the sponsor and will be documented in a memorandum.

### Dissemination plans {31a}

The TSC is responsible for all publications. The scientific integrity of the project requires that the data from all TOTO sites be analyzed study-wide and reported as such. Thus, an individual center is not expected to report the data collected from its center alone. All presentations and publications are expected to protect the integrity of the major objective(s) of the study; data that break the blind will not be presented prior to the release of mainline results. Primary outcome papers in peer-reviewed publications and other study papers, abstracts, and presentations are planned. The study results will be released to the participating physicians, referring physicians, patients, and the general medical community.

## Discussion

TOTO follows a public contract by the G-BA, the highest decision-making body for joint self-government in the German healthcare system. TOTO evaluates if a tonsillotomy is not inferior to a tonsillectomy if tonsil surgery is indicated for recurrent acute tonsillitis. The findings will likely have a high impact on clinical management strategies in Germany and may be beyond Germany. Based on the results, the G-BA will probably decide if tonsillotomy because a standard procedure or alternative to tonsillectomy for the treatment of recurrent acute tonsillitis. Using randomization in this study design, we aim to limit confounders as much as possible. Nevertheless, as the effect of tonsillotomy versus tonsillectomy is obvious to the participant and involved physicians, blinding was not feasible. Therefore, it seems for us to be very important that the involved biostatistician will be blinded until database lock and that the assessment of the primary endpoint and secondary endpoints is following a strict evaluation algorithm. A strength but also a challenge is the long follow-up period of 24 months. To sustain the compliance over such a long period, a concept with only a little interference for the participants/parents was chosen. Visits in the study are not necessary if the follow-up is uneventful. By use of the TOTO app, the follow-up parameters can be delivered within seconds to a few minutes.

Two important issues cannot be solved by the TOTO trial. First, the definition of recurrent acute tonsillitis is mainly based on clinical diagnosis, i.e., has a subjective component. So far, clear objective parameters are missing [15]. Furthermore, the indication for tonsil surgery in case of recurrent acute tonsillitis is internationally seen very controversially. We followed with our very strict inclusion criteria the German clinical guideline [4]. This guideline, in turn, follows the strict criteria based on the milestone publications of Paradise et al. [16, 17]. Second, TOTO compares two methods of tonsil surgery. A comparison to conservative management is not performed. To ask this question, the NAtional randomised controlled Trial of Tonsillectomy IN Adults (NATTINA) study was designed [5]. NATTINA recruitment ended successfully and we look forward to the results. As the TOTO 10-question survey in case of sore throat covers all questions asked also in the NATTINA questionnaire and because NATTINA also used the TOI-14 and the SF-12 as secondary outcome criteria, a common pooled analysis will be feasible. Such a pooled analysis with gain additional information on the comparison of tonsillotomy versus conservative treatment as in NATTINA only tonsillectomy is performed as a surgical method.

## Trial status

Protocol version number: V 1.1; date: 08-May-2020; date of planned recruitment begin:01-September-2020; the approximate date when recruitment will be completed: 01-September 2022.

## Supplementary Information

**Additional file 1.** Toto consent care givers.

**Additional file 2.** Toto consent 3–6 years.

**Additional file 3.** Toto consent 7–11 years.

**Additional file 4.** Toto consent 12–15 years.

**Additional file 5.** Toto consent 16+ years.

## Data Availability

The TSC and the investigators will have access to the final trial dataset. We disclose contractual agreements that limit such access for investigators.

## Abbreviations

AE: Adverse event
DMC: Data monitoring committee
eCRF: Electronic case report form
EDC: Electronic data capture
GCP: Good clinical practice
IQWiG: German Institute for Quality and Efficiency in Health Care (Institut für Qualität und Wirtschaftlichkeit im Gesundheitswesen)
G-BA: Federal Joint Committee (Gemeinsamer Bundesausschuss)
IIT: Intention to treat
NRS: Numeric rating scale
PP: Per protocol
SAE: Serious adverse event
SAP: Statistical analysis plan
TAHSI: Tonsil and Adenoid Health Status Instrument
TOI: Tonsillectomy Outcome Inventory
TOTO: Scronym of the trial
TSC: Trial steering committee

## References

[1] Windfuhr JP, Alizoti P, Hendricks C. Regional variability of hemorrhage following tonsil surgery in 1,520,234 cases. Eur Arch Otorhinolaryngol. 2020. 10.1007/s00405-020-06080-x Online ahead of print. 10.1007/s00405-020-06080-x 32451670

[2] Nolting HD, Zich K, Deceknbach B (2013). Faktencheck Gesundheit. Entfernung der Gaumenmandeln bei Kindern und Jugendlichen.

[3] Sarny S, Habermann W, Ossimitz G, Stammberger H (2013). What lessons can be learned from the Austrian events?. ORL J Otorhinolaryngol Relat Spec.

[4] AWMF Leitlinie (2016). Therapie entzündlicher Erkrankungen der Gaumenmandeln - Tonsillitis.

[5] Rubie I, Haighton C, O'Hara J, Rousseau N, Steen N, Stocken DD (2015). The NAtional randomised controlled Trial of Tonsillectomy IN Adults (NATTINA): a clinical and cost-effectiveness study: study protocol for a randomised control trial. Trials..

[6] Tonsillotomie bei rezidivierender akuter Tonsillitis und bei Hyperplasie der Tonsillen. Köln; 2017. Contract No.: N15-11; https://www.iqwig.de/en/projects-results/projects/non-drug-interventions/n-projekte/n15-11-tonsillotomy-for-recurrent-acute-tonsillitis-and-for-hyperplasia-of-the-tonsils.7133.html.

[7] Wong Chung J, van Benthem PPG, Blom HM (2018). Tonsillotomy versus tonsillectomy in adults suffering from tonsil-related afflictions: a systematic review. Acta Otolaryngol.

[8] Lock C, Wilson J, Steen N, Eccles M, Mason H, Carrie S (2010). North of England and Scotland Study of Tonsillectomy and Adeno-tonsillectomy in Children(NESSTAC): a pragmatic randomised controlled trial with a parallel non-randomised preference study. Health Technol Assess.

[9] Witsell DL, Orvidas LJ, Stewart MG, Hannley MT, Weaver EM, Yueh B, Smith TL, Goldstein NA, TO TREAT Study Investigators (2008). Quality of life after tonsillectomy in adults with recurrent or chronic tonsillitis. Otolaryngol Head Neck Surg.

[10] Skevas T, Klingmann C, Plinkert PK, Baumann I (2012). Development and validation of the Tonsillectomy Outcome Inventory 14. HNO..

[11] Steinbichler T, Bender B, Blassnigg E, Riechelmann H (2014). Evaluation of a German version of the tonsil and adenoid health status instrument. J Otolaryngol Head Neck Surg.

[12] Ware J, Kosinski M, Keller SD (1996). A 12-Item Short-Form Health Survey: construction of scales and preliminary tests of reliability and validity. Medical Care.

[13] Stewart MG, Friedman EM, Sulek M, de Jong A, Hulka GF, Bautista MH (2001). Validation of an outcomes instrument for tonsil and adenoid disease. Arch Otolaryngol Head Neck Surg.

[14] Seethaler A, Rudack C, Spiekermann C (2019). Structured literature review of patient-reported outcome (PRO) instruments in adult tonsillectomy or tonsillotomy. Health Qual Life Outcomes.

[15] Geissler K, Bohne S, Siggel R, Sachse S, Kiehntopf M, Bauer M (2014). Preoperative serum pattern analysis to predict the outcome of tonsillectomy in adults with chronic tonsillitis. Eur Arch Otorhinolaryngol.

[16] Paradise JL, Bluestone CD, Bachman RZ, Colborn DK, Bernard BS, Taylor FH, Rogers KD, Schwarzbach RH, Stool SE, Friday GA, Smith IH, Saez CA (1984). Efficacy of tonsillectomy for recurrent throat infection in severely affected children. Results of parallel randomized and nonrandomized clinical trials. N Engl J Med.

[17] Paradise JL (2008). Indications for tonsillectomy: setting the bar high enough. Arch Otolaryngol Head Neck Surg.

