# Supplementary material for: Tonsillectomy versus tonsillotomy for recurrent acute tonsillitis in children and adults (TOTO): study protocol for a randomized non-inferiority trial
Source: Trials. 2021 Jul 22;22:479. doi: 10.1186/s13063-021-05434-y (PMC8296750; doi:10.1186/s13063-021-05434-y)
Supplement: Supplementary file 1 — Additional file 1. Toto consent care givers. [file 13063_2021_5434_MOESM1_ESM.pdf]

INFORMATION FÜR ELTERN UND SORGEBERECHTIGTE

Informationsblatt und Einwilligungserklärung zur Studie:

Tonsillektomie versus Tonsillotomie bei Kindern und Erwachsenen mit rezidivierender akuter Tonsillitis: Eine kontrollierte, randomisierte Nichtunterlegenheits-Studie

Kurztitel:	Toto
Prüfplan-Nummer:	UMG20775
DRKS-Nummer:	DRKS 00020283
Version:	1.0 vom 17.04.2020

Name, Anschrift und Telefonnummer des Studienarztes (Prüfarztes) Ihres Kindes:

Inhalt der Patienteninformation

INFORMATIONEN ZUM STUDIENABLAUF **5**
Organisation und Verantwortlichkeit 5
Kontaktdaten Ihres behandelnden Studienzentrums (Prüfzentrum) 6
Warum wird die Studie durchgeführt? 6
Zielsetzung der Studie 7
Wie ist der Ablauf der Studie und was müssen Sie wissen? 7
Welche Risiken bestehen für Ihr Kind? 9
Behandlungsmöglichkeiten außerhalb der Studie 10
Wer darf bei dieser Studie nicht teilnehmen? 10
Was kostet die Studie? Erhält mein Kind Geld zurück? 10
Ist Ihr Kind während der klinischen Studie versichert? 10
Mitteilung neuer Erkenntnisse während der klinischen Studie 11
Öffentliche Registrierung der Studie und Veröffentlichungen 11
Beendigung der klinischen Studie 11
Datenschutz 12
Für weitere Fragen 12
Persönliche Notizen 13
EINVERSTÄNDNISERKLÄRUNG **14**
Toto: 14
DATENSCHUTZ **16**

Sehr geehrte Eltern, sehr geehrte Sorgeberechtigte,

im Folgenden erhalten Sie einen Überblick über die Inhalte der Patienteninformation zu der TOTO-Studie. Dieser soll einer ersten Orientierung dienen, kann jedoch nicht die Inhalte der ausführlichen Patienteninformation ersetzen. Wir bitten Sie daher, die Patienteninformation vollständig und gewissenhaft durchzulesen.

- Mit der nachfolgenden Patienteninformation klären wir Sie über die Teilnahme Ihres Kindes an der klinischen Studie „TOTO" auf. Die Teilnahme Ihres Kindes ist freiwillig und setzt Ihr schriftliches Einverständnis voraus.

- Die vorliegende Studie beschäftigt sich mit Operationsverfahren bei wiederholter akuter Mandelentzündung (Fachbegriff: rezidivierende akute Tonsillitis).

- Die Studie wird vom Universitätsklinikum Jena in Zusammenarbeit mit der Deutschen Gesellschaft für Hals-Nasen-Ohren-Heilkunde, Kopf- und Hals-Chirurgie und dem Deutschen Berufsverband der Hals-Nasen-Ohrenärzte sowie dem Studienzentrum der Universitätsmedizin Göttingen organisiert.

- Wir möchten im Auftrag des Gemeinsamen Bundesausschusses (G-BA) mit Hilfe dieser Studie untersuchen, wie sich zwei unterschiedliche Operationsverfahren auf die Erkrankung Ihres Kindes auswirkt. Der G-BA ist das höchste Beschlussgremium der gemeinsamen Selbstverwaltung im deutschen Gesundheitswesen.

- Einem Teil der Patienten werden **die Gaumenmandeln vollständig entfernt (Fachbegriff: Tonsillektomie)**. Einem anderen Teil werden **die Gaumenmandeln teilweise entfernt (Fachbegriff: Tonsillotomie).** Bislang ist es Standard, eine vollständige Entfernung der Gaumenmandeln vorzunehmen. Der G-BA möchte klären, ob eine teilweise Entfernung nicht ausreichend ist.

- Welches operative Verfahren zum Einsatz kommt, wird im Rahmen der Studienzuteilung nach zuvor festgelegten Zufallsverfahren, vergleichbar mit dem Werfen einer Münze, entschieden.

- Die **Studie dauert** zwei Jahre.

- Wenn Sie nicht möchten, dass Ihr Kind an der Studie teilnimmt, wird Ihr behandelnder Arzt eine Therapie für Ihr Kind wählen, das es auch bei einer Studienteilnahme bekommen hätte. Sie und Ihr Kind können die **Teilnahme an der Studie jederzeit** auch während der Studie **beenden**, ohne dass Ihnen oder Ihrem Kind dadurch Nachteile entstehen.

- Ihr Kind kann NICHT an der Studie teilnehmen, sollten es gleichzeitig **an anderen klinischen Studien teilnehmen** oder innerhalb von 4 Wochen vor Studienbeginn

teilgenommen haben.

- Ihr Kind kann ebenfalls NICHT teilnehmen, sollte es **schwanger** sein oder **stillen**.

- Durch die Teilnahme an dieser klinischen Studie entstehen Ihnen gegenüber der üblichen Therapie **keine zusätzlichen Kosten.**

- Alle Patienten die an der Studie teilnehmen sind **versichert.**

- Bei Rücktritt von der Studie können auf Wunsch bereits gewonnene Daten vernichtet werden. Sie können sich bei der Rücknahme der Einwilligung entscheiden, ob Sie mit der Auswertung des Materials bzw. der Studiendaten Ihres Kindes einverstanden sind oder nicht. Sollten Sie zu einem späteren Zeitpunkt Ihre Entscheidung ändern wollen, setzen Sie sich bitte mit dem Studienarzt in Verbindung.

  Im Rahmen der Studie werden **persönliche Daten** Ihres Kindes erhoben und **gespeichert**. Dies erfolgt in **pseudonymisierter Form.** Eine Weitergabe erfolgt nur in anonymisierter Form. Pseudonymisierung bedeutet, dass Ihrem Kind im Rahmen der Studie eine Nummer als Studienteilnehmer zugewiesen wird, die einen Rückschluss auf die Identität Ihres Kindes ausschließt oder wesentlich erschwert. Die Zuordnung zwischen Pseudonymisierungsnummer und den Namen Ihres Kindes kann nur an dem behandelnden Studienzentrum erfolgen. Bei einer Anonymisierung wäre auch diese Zuordnung nicht mehr möglich.

Sehr geehrte Eltern, sehr geehrter Sorgeberechtigte,

Ihr Einverständnis zur Teilnahme Ihres Kindes an einer klinischen Studie

wir möchten Sie fragen, ob Sie bereit sind, Ihr Kind an der nachfolgend beschriebenen klinischen Studie teilnehmen zu lassen.

Solche Studien dienen der Forschung in der Medizin. Sie sind notwendig, um genauer herauszufinden, ob und wie gut neue Medikamente wirken oder – wie im vorliegenden Fall - wie bestimmte medizinische Verfahren wirken und wie gut sie vertragen werden.

Die Teilnahme Ihres Kindes an dieser klinischen Studie ist freiwillig. Es wird in diese Studie also nur dann einbezogen, wenn Sie dazu schriftlich Ihre Einwilligung erklären. Sofern Sie Ihr Kind nicht an der klinischen Studie teilnehmen lassen wollen oder Ihr Kind später aus ihr Ausscheiden lassen möchten, entsteht Ihrem Kind daraus keine Nachteile.

Sie wurden bereits auf die geplante Studie angesprochen. Der nachfolgende Text soll Ihnen die Ziele und den Ablauf erläutern. Anschließend wird ein behandelnder Studienarzt (Prüfarzt) das Aufklärungsgespräch mit Ihnen führen. Bitte zögern Sie nicht, alle Punkte anzusprechen, die Ihnen unklar sind. Sie haben danach ausreichend Bedenkzeit, um mit Ihrem Kind über die Studie zu sprechen und über die Teilnahme zu entscheiden.

Informationen zum Studienablauf

Organisation und Verantwortlichkeit

Organisation und Verantwortlichkeit

Diese klinische Studie wird multizentrisch in ca. 20 Zentren in Deutschland durchgeführt; es sollen insgesamt 554 Patienten daran teilnehmen. Die Studie wird von der Universitätsmedizin Jena in Zusammenarbeit mit dem Studienzentrum Göttingen organisiert. Finanziert wird die Studie durch den gemeinsamen Bundesausschuss (G-BA). Die operativen Verfahren, die in dieser Studie betrachtet werden, werden von der Krankenkasse vergütet.

Die Studie wurde von der zuständigen Ethikkommission zustimmend bewertet.

Kontaktdaten Ihres behandelnden Studienzentrums (Prüfzentrum)

Kontaktdaten
Ihres behandelnden Studienzentrums (Prüfzentrum

**Prüfzentrum** (Stempel):	**Leiter/in der klinischen Studie:**
	Herr Prof. Guntinas-Lichius<br><br>HNO-Klinik<br>Universitätsklinikum Jena<br>Am Klinikum 1<br>07747 Jena<br><br>Telefon: 03641-9-329301<br>E-Mail:<br>Orlando.Guntinas@med.uni-jena.de

Warum wird die Studie durchgeführt?

Operative Verfahren bei wiederholten akuter Mandelentzündung

Warum wird die Studie durchgeführt

Zur Entfernung der Gaumenmandeln finden derzeit zwei operative Verfahren standardmäßig in der Medizin Anwendung. Bei dem Einen handelt es sich um eine teilweise Entfernung der Gaumenmandeln (Tonsillotomie), bei dem Anderen um eine vollständige Entfernung der Gaumenmandeln (Tonsillektomie). Die teilweise Entfernung wird vor allem bei kleinen Kindern mit Atemproblemen vorgenommen. Die vollständige Entfernung der Gaumenmandeln ist dagegen bislang Standard zur Behandlung der wiederholten akuten Mandelentzündung (rezidivierende akute Tonsillitis).

Bislang konnte noch nicht geklärt werden, ob bei Patientinnen und Patienten mit immer wiederkehrenden akuter Mandelentzündungen, bei der der behandelnde Arzt, einen chirurgischen Eingriff empfehlen würde, eine Tonsillotomie gegenüber einer Tonsillektomie nicht unterlegen ist. Mit der Durchführung dieser Studie soll diese Fragestellung geklärt werden.

Kann ich entscheiden, welches Verfahren bei meinem/unserem Kind zur Anwendung kommt?

Einschluss in einem bestimmten operativen Arm

Im Rahmen dieser Studie wird die Tonsillotomie mit der Tonsillektomie verglichen, um Wirkungen und Nebenwirkungen der Verfahren besser beurteilen zu können. Beide Verfahren sind für die Therapie bei wiederkehrenden akuten Mandelentzündungen zugelassen. Deshalb werden alle Patienten, die an der Studie teilnehmen, in zwei Gruppen eingeteilt. Die eine Gruppe erhält eine Ton-

sillotomie, die andere Gruppe eine Tonsillektomie.

Zu welcher Gruppe Ihr Kind im Falle der Teilnahme gehört, wird nach Zufall entschieden, vergleichbar mit dem Werfen einer Münze. Die Wahrscheinlichkeit, dass Ihr Kind eine Tonsillotomie erhalten, beträgt 50%. Der Arzt und Sie wissen aber in welcher Gruppe Ihr Kind ist.

Zielsetzung der Studie

Ziel der Studie

Das Hauptziel der Studie ist es, zu zeigen, dass die Tonsillotomie nicht weniger gut hilft als die Tonsillektomie.

Weitere Ziele

Weitere Ziele sind u.a.:

- Die systematische Erfassung von Nebenwirkungen und Anzahl der Tage, an denen Halsschmerzen nach der Operation auftreten

Wie ist der Ablauf der Studie und was müssen Sie wissen?

Voruntersuchung

Voruntersuchung

Wenn Ihr Kind an der Studie teilnimmt, wird zuerst die Vorgeschichte der Erkrankung abgefragt und es wird umfassend ärztlich untersucht. Dazu gehören Fragen zu seiner Krankheitsgeschichte und auch medizinische Untersuchungen (bspw. Gewicht, Größe). Ob Ihr Kind an der klinischen Studie teilnehmen kann, hängt von den Ergebnissen dieser Voruntersuchung ab.

Ablauf der Studie und Untersuchungen im Laufe der Studie

Wenn Sie und Ihr Kind der Studienteilnahme zugestimmt haben und die Eingangsuntersuchungen durchgeführt wurden, erfolgt die zufällige (randomisierte) Zuteilung in eine der beiden Gruppen (= Behandlungsarme - wie auf der vorherigen Seite beschrieben).

• Bei der Operation werden Ihrem Kind die Gaumenmandeln teilweise oder vollständig entfernt

• Über einen Zeitraum von 24 Monaten werden wir Ihnen bzw. Ihrem Kind wöchentlich Fragen stellen, die

- Die Häufigkeit und Schwere der Halsschmerzen Ihres Kindes betreffen.
- Fragen zur Lebensqualität Ihres Kindes beinhalten.

Hierzu werden wir Ihnen die Möglichkeit geben, die Fragen mithilfe einer App, über eine Webseite oder eines Tagebuchs zu beantworten.

Zusätzlich werden wir Sie und insbesondere Ihr Kind in regelmäßigen Abständen (und zwar alle sechs Monate über einen Zeitraum von zwei Jahren) telefonisch kontaktiert, um Ihnen, bzw. Ihrem Kind Fragen zu seinem Wohlbefinden zu stellen. Bei eventuellen Rückfragen möchte das Studienzentrum (Prüfzentrum) Sie bzw. Ihr Kind auch gerne zwischendurch einmal kontaktieren dürfen.

Dauer der Studie

Dauer der Studie

Die Operation und die danach folgende Behandlung dauert nur wenige Tage. Nach der Operation wollen wir jedoch über einen Zeitraum von zwei Jahren wissen, ob Ihr Kind noch weiterhin regelmäßig Halsschmerzen hat und falls ja wie stark diese Halsschmerzen sind.

Zeitlicher Ablauf der Studie

Der zeitliche Ablauf der Studie ist wie folgt vorgesehen:

Bezeichnung/Monat	Screening und Baseline[a]	Operation[a]	wöchentliche Patientendatenerhebungen (bis Monat 24)[b]	FU Monat[b] 6, 12, 18, 24
Ein-Ausschlusskriterien	X			
Patientendaten	X			
Einwilligungserklärung	X			
Randomisierung	X			
OP Daten		X		
Blutungen / UEs		X	X	X
Anzahl Tage Halsschmerzen / NRS	X		X	X
STAR (Wenn Halschmerzen vorliegen)*			X	X
TAHSI*	X			X
TOI*	X			X
SF-12 und im STAR*	X			X

[a] = am Zentrum
[b] = Datenerhebung durch Patient [ggf. Weiterleitung Daten (bspw. Tagebuch)]
* Bei Weiterleitung der Daten: Arbeitsanfall am Zentrum und zentrale Datenerfassung

Was müssen Sie bei einer Studienteilnahme Ihres Kindes beachten?

- Da Ihr Kind nach der Entlassung bei normalem Verlauf nicht mehr in der Klinik vorstellig werden wird und nach der Abheilung auch in der Regel nicht mehr Ihren HNO-Arzt oder Kinderarzt besuchen wird, ist es wichtig, dass Sie und Ihr Kind die Fragen nach Hallschmerzen wöchentlich selbst beantworten. Das wollen wir Ihnen so einfach wie möglich machen.
- Wenn Ihrem in dieser Patienteninformation genanntem behandelnden Arzt auffällt, dass Sie die Fragen nicht regelmäßig beantworten, kann es sein, dass der Arzt bei Ihnen nachfragen wird und Sie bitten, die Fra-

gen, regelmäßig zu erfassen.

- Teilen Sie dem Studienpersonal alle Erkrankungen und Verletzungen, einschließlich Verschlechterungen des Gesundheitszustands, die während der Studie bei Ihrem Kind auftreten, mit.

Was bringt es Ihrem Kind, wenn es an der Studie teilnimmt und Sie einwilligen?

Tatsächlich geht der Beschluss des G-BA, diese Studie vornehmen zu lassen, ursprünglich auf eine Patientenanfrage zurück. Eltern haben beim G-BA angefragt, ob es denn tatsächlich notwendig sei, immer die gesamten Mandeln zu entfernen statt zu einem Teil zu entfernen. Darauf hat der G-BA eine wissenschaftliche Prüfung veranlasst, die ergab, dass dies eben bei Patienten, die wegen wiederholten Mandelentzündungen operiert werden müssten, unklar sei. Durch die Teilnahme Ihres Kindes können Sie helfen diese Frage zu klären und dazu beitragen wie zukünftig Kinder und Erwachsene mit dieser Erkrankung am besten behandelt werden.

Welche Risiken bestehen für Ihr Kind?

Risiken

Jede Operation, so auch eine Mandeloperation, ist mit bestimmten Risiken versehen. Dies hat nichts mit einer Studienteilnahme zu tun. Diese Risiken bestehen auch, wenn eine Mandeloperation außerhalb einer Studie vorgenommen wird. Durch die Studienteilnahme ergibt sich kein zusätzliches Risiko.

Bei einer teilweisen Entfernung der Gaumenmandeln (Tonsillotomie) können Nachblutung, Blutung, Sprachklangänderung (z.B. offenes Näseln), Schluckbeschwerden, Zahn-, Zungen-, Schleimhautschädigung und Überschlucken in die Nase, als Risiken auftreten.

Bei einer kompletten Entfernung der Gaumenmandeln (Tonsillektomie) können Nachblutung (auch bis zu 14 Tage nach Operation) mit möglicherweise letalem Ausgang, Blutung, Sprachklangänderung (z.B. offenes Näseln), Schluckbeschwerden, Zahn-, Zungen-, Schleimhautschädigung und Überschlucken in die Nase, als Risiken auftreten.

Bezüglich der Operation und den damit verbundenen speziellen Risiken werden Sie und Ihr Kind separat informiert und aufgeklärt.

Behandlungs-möglichkeiten außerhalb der Studie

Behandlungsmöglichkeiten außerhalb der Studie

Wenn Sie und Ihr Kind nicht an der Studie teilnehmen möchten, wird Ihr Kind natürlich außerhalb der Studie behandelt.

Schwangere und stillende Frauen und Mädchen dürfen nicht teil-nehmen

Wer darf bei dieser Studie nicht teilnehmen?

Ihr Kind darf nicht teilnehmen, wenn es gleichzeitig an anderen Studien teilnimmt oder vor kurzem teilgenommen hat.

Sollte Ihr Kind schwanger sein oder stillen, darf es ebenfalls nicht an der klinischen Studie teilnehmen.

Was kostet die Studie? Erhält mein Kind Geld zurück?

Durch die Teilnahme an dieser Studie entstehen für Ihr Kind **keine** zusätzlichen Kosten. Fahrtkosten zur Praxis/Klinik als Studienzentrum werden nicht erstattet.

Versicherungs-schutz während der Studie

Ist Ihr Kind während der klinischen Studie versichert?

Ihr Kind ist während der Studie gegen eventuelle Gesundheitsschäden durch die Studienteilnahme versichert. Das betrifft auch die Frage, was geschehen muss, wenn Sie den Verdacht haben, dass die Studie bei Ihrem Kind einen Gesundheitsschaden verursacht haben könnte.

**Name der Versicherung:**	HDI Global SE
**Anschrift der Versicherung:**	Am Schönenlamp 45<br>40559 Düsseldorf
**Versicherungsnummer:**	65964770103017
**Telefon:**	0211 7482-176

Mitteilungspflicht an den Versiche-rer

Wenn Sie vermuten, dass durch die Teilnahme an der klinischen Studie die Gesundheit Ihres Kindes geschädigt oder bestehende Leiden verstärkt wurden, müssen Sie dies unverzüglich dem Versicherer direkt anzeigen, gegebenenfalls mit Unterstützung durch Ihren behandelnden Arzt (Prüfarzt), um Ihren Versicherungsschutz nicht zu gefährden. Sofern Ihr Prüfarzt Sie dabei unterstützt, erhalten Sie eine Kopie der Meldung. Sofern Sie Ihre Anzeige direkt an den Versicherer richten, informieren Sie bitte zusätzlich Ihren Prüfarzt.

Mitteilung neuer Erkenntnisse während der klinischen Studie

Sie werden über neue Erkenntnisse, die in Bezug auf die Operationsmethode bekannt werden und die für Ihre Bereitschaft zur weiteren Teilnahme Ihres Kindes wesentlich sein können, informiert. Auf dieser Basis können Sie dann Ihre Entscheidung zur weiteren Teilnahme an dieser klinischen Studie überdenken.

Öffentliche Registrierung der Studie und Veröffentlichungen

Wo sind Informationen über diese Studie öffentlich verfügbar?

Studienregister

Diese Studie ist in einem öffentlich zugänglichen Register eingetragen. Dieses Register heißt Deutsches Register Klinischer Studien. Die Studie ist mit der Nummer DRKS00020823 dort registriert und ist unter der folgenden Webadresse verfügbar:

https://www.drks.de/drks_web/

In diesem öffentlichen Register finden Sie allgemeine Informationen über die Studie. Diese Website enthält aber keine Informationen, die zur Identifikation Ihres Kindes führt.

Veröffentlichungen und Publikationen

Die Ergebnisse dieser Studie sollen veröffentlicht werden, z.B. in wissenschaftlichen Zeitschriften und auf Konferenzen. Diese Veröffentlichungen werden keinerlei persönliche Daten enthalten, die Rückschlüsse auf Ihr Kind ermöglichen. Zudem ist es möglich, dass die Forschungsergebnisse kommerziell genutzt, z.B. patentiert werden. An einem möglichen kommerziellen Nutzen werden Sie und Ihr Kind nicht beteiligt.

Beendigung der klinischen Studie

Beendigung der klinischen Studie

.

Sie und Ihr Kind können die klinische Studie jederzeit, ohne Angabe von Gründen beenden, ohne dass Ihnen oder Ihrem Kind dadurch Nachteile entstehen.

Unter gewissen Umständen ist es aber auch möglich, dass der Prüfarzt oder der Sponsor entscheidet, die Teilnahme Ihres Kindes an der klinischen Studie vorzeitig zu beenden, ohne dass Sie auf die Entscheidung Einfluss haben. Die Gründe hierfür können z. B. sein:

- Die weitere Teilnahme Ihres Kindes an der klinischen Studie ist ärztlich nicht mehr vertretbar;
- die gesamte klinische Studie wird abgebrochen.

Zuständige Ethikkommissionen können die Studie ebenfalls jederzeit beenden.

Sofern Sie sich dazu entschließen, Ihr Kind vorzeitig aus der klinischen Studie ausscheiden zu lassen oder seine Teilnahme aus einem anderen der genannten Gründe vorzeitig beendet wird, ist es für die eigene Sicherheit wichtig, dass Ihr Kind sich einer empfohlenen abschließenden Kontrolluntersuchung unterzieht. Der Prüfarzt wird mit Ihnen besprechen, wie und wo die weitere

Behandlung Ihres Kindes stattfindet.

Datenschutz

Pseudonymisierung bedeutet verschlüsseltes Speichern von persönlichen Daten

Während der klinischen Studie werden medizinische Befunde und persönliche Informationen von Ihrem Kind erhoben und in dem Prüfzentrum in einer persönlichen Akte niedergeschrieben oder elektronisch gespeichert. Die für die klinische Studie wichtigen Daten werden zusätzlich in pseudonymisierter Form gespeichert, ausgewertet und gegebenenfalls weitergegeben.

Pseudonymisiert bedeutet, dass keine Angaben von Namen oder Initialen verwendet werden, sondern nur ein Nummern- und/oder Buchstabencode.

Die Daten sind gegen unbefugten Zugriff gesichert. Eine Entschlüsselung erfolgt nur unter den vom Gesetz vorgeschriebenen Voraussetzungen.

Einzelheiten, insbesondere zur Möglichkeit eines Widerrufs, entnehmen Sie bitte der Einwilligungserklärung, die im Anschluss an diese Informationsschrift abgedruckt ist.

Für weitere Fragen

Beratungsgespräche in dem Prüfzentrum

Sie können bei jeder Gelegenheit in Ihrem Prüfzentrum Fragen stellen. Die Adresse finden Sie auf der Seite 1 dieser Informationsschrift.

Sie erhalten eine Kopie dieser Informationsschrift und der unterschriebenen Einverständniserklärung sowie der Versicherungsbedingungen.

Unabhängig von Ihrer Entscheidung oder der Entscheidung Ihres Kindes über eine Teilnahme an der Studie, wünschen wir Ihnen für den weiteren Behandlungsverlauf Ihres Kindes alles Gute.

Falls Sie keine weiteren Fragen haben und sich für Ihr Kind zur Teilnahme an dem wissenschaftlichen Projekt entschieden haben, unterzeichnen Sie bitte die beiliegende Einverständniserklärung.

Persönliche Notizen

Einverständniserklärung

Toto: Tonsillektomie versus Tonsillotomie bei Kindern und Erwachsenen mit rezidivierender akuter Tonsillitis: Eine kontrollierte, randomisierte Nichtunterlegenheits-Studie

**Prüfzentrum** *(Stempel):*	**Prüfarzt:**
	Name: ____________________
	Telefon: ____________________

Name des Patienten in Druckbuchstaben

/ /

geboren am

/

Zentrums-Nr. / Patienten-Nr.

Ich bin/wir sind in einem persönlichen Gespräch durch den Prüfarzt

Name der Ärztin / des Arztes

ausführlich und verständlich über den chirurgischen Eingriff sowie über Wesen, Bedeutung, Risiken und Tragweite der klinischen Studie aufgeklärt worden. Ich habe/wir haben darüber hinaus den Text der Patienteninformation sowie die hier nachfolgend abgedruckte Datenschutzerklärung gelesen und verstanden. Ich hatte/wir hatten die Gelegenheit, mit dem Prüfarzt über die Durchführung der klinischen Studie zu sprechen. Alle unsere Fragen wurden zufrieden stellend beantwortet.

Möglichkeit zur Dokumentation zusätzlicher Fragen seitens des Patienten oder sonstiger Aspekte des Aufklärungsgesprächs:

____

____

____

____

Ich hatte/wir hatten ausreichend Zeit, mich/uns zu entscheiden.

Mir/uns ist bekannt, dass ich/wir jederzeit und ohne Angabe von Gründen meine/unsere Einwilligung zur Teilnahme an der Studie zurückziehen können (mündlich oder schriftlich), ohne dass meinem/unserem Kind daraus Nachteile für die medizinische Behandlung entstehen.

Datenschutz

Mir/uns ist bekannt, dass bei dieser klinischen Studie personenbezogene Daten, insbesondere medizinische Befunde über mein/unser Kind erhoben, in pseudonymisierter Form gespeichert und ausgewertet werden. Die personenbezogenen Daten werden nur nach entsprechender Anonymisierung an Dritte weitergegeben. Die Verwendung der Angaben über seine Gesundheit erfolgt nach gesetzlichen Bestimmungen und setzt vor der Teilnahme an der klinischen Studie folgende freiwillig abgegebene Einwilligungserklärung voraus, das heißt ohne die nachfolgende Einwilligung kann mein/unser Kind nicht an der klinischen Studie teilnehmen.

1. Ich/Wir erkläre/n uns damit einverstanden, dass im Rahmen dieser klinischen Studie personenbezogene Daten, insbesondere Angaben über die Gesundheit, über mein/unser Kind erhoben und in Papierform sowie auf elektronischen Datenträgern im

   ....

   (Prüfzentrum hier eintragen)

   aufgezeichnet werden. Soweit erforderlich, dürfen die erhobenen Daten pseudonymisiert (verschlüsselt) weitergegeben werden:

   a) an den Sponsor, die HNO-Klinik Universitätsklinikum Jena oder eine von dieser beauftragten Stelle zum Zwecke der wissenschaftlichen Auswertung,

   b) an das Studienzentrum der Universitätsmedizin Göttingen zur Organisation aller für die klinische Studie relevanten Tätigkeiten,

   c) im Falle unerwünschter Ereignisse: im Auftrag des Sponsors an das Studienzentrum der Universitätsmedizin Göttingen und an die jeweils zuständige Ethik-Kommission

   d) Außerdem erkläre/n ich/wir mich/uns damit einverstanden, dass autorisierte und zur Verschwiegenheit verpflichtete Beauftragte des Sponsors in die beim Prüfarzt vorhandenen personenbezogenen Daten meines/unseres Kindes, insbesondere seine Gesundheitsdaten, Einsicht nehmen, soweit dies für die Überprüfung der ordnungsgemäßen Durchführung der Studie notwendig ist.

2. Die Einwilligung zur Erhebung und Verarbeitung der personenbezogenen Daten, insbesondere der Angaben über die Gesundheit, kann von mir/uns jederzeit widerrufen werden. Ich bin/Wir sind bereits darüber aufgeklärt worden, dass ich/wir jederzeit die Teilnahme meines/unseres Kindes an der klinischen Studie beenden können. Im Fall eines solchen Widerrufs der Einwilligung, an der Studie teilzunehmen, erkläre ich mich/wir uns

   ☐ **damit einverstanden**, dass die bis zu diesem Zeitpunkt gespeicherten Daten ohne Namensnennung/Personenbezug weiterhin verwendet werden dürfen

   ☐ **nicht damit einverstanden**, dass die bis zu diesem Zeitpunkt gespeicherten Daten nicht weiterhin verwendet werden. Die Daten müssen in diesem Fall im Rahmen der

technischen Möglichkeiten gelöscht, bzw. anonymisiert werden.

3. Ich/wir erkläre/n uns damit einverstanden, dass die Daten nach Beendigung oder Abbruch der Studie mindestens zehn Jahre aufbewahrt werden. Danach werden die personenbezogenen Daten gelöscht, soweit nicht gesetzliche Aufbewahrungsfristen entgegenstehen.

   Ich bin/wir sind über folgende gesetzliche Regelung informiert: Falls wir unsere Einwilligung, an der Studie teilzunehmen, widerrufe, müssen alle Stellen, die personenbezogenen Daten, insbesondere Gesundheitsdaten, gespeichert haben, unverzüglich prüfen, inwieweit die gespeicherten Daten für die in Nr. 3 a) bis b) genannten Zwecke noch erforderlich sind.

   Nicht mehr benötigte Daten sind unverzüglich zu löschen.

4. Ich bin/wir sind damit einverstanden, dass mein/unser Kind innerhalb des Nachuntersuchungszeitraums regelmäßig (und zwar alle sechs Monate über einen Zeitraum von zwei Jahren) und eventuell zwischenzeitlich, telefonisch kontaktiert werden darf, um Informationen über dessen Wohlbefinden zu erhalten, durch

   ....

   (Prüfzentrum hier eintragen)

5. Ich bin/wir sind damit einverstanden, dass der Hausarzt/niedergelassene behandelnde Arzt meines/unseres Kindes

   ....
   (Namen hier eingetragen)

   über die Teilnahme an der klinischen Studie informiert wird (falls nicht gewünscht, bitte streichen).

6. Der Verantwortliche für die Datenerhebung dieser klinischen Studie ist:

   Universitätsmedizin Göttingen, Studienzentrum UMG, erreichbar über:

   Von Bar Str. 2/4, 370775 Göttingen, Tel. 0551-39-60812

7. Der für diese klinische Studie verantwortliche Datenschutzbeauftragte ist:

Datenschutzbeauftragter des Universitätsklinikums Jena

Zentrum für Gesundheits-und Sicherheitsmanagement,

Beauftragte für Datenschutz des Universitätsklinikum Jena

Adresse:	Bachstraße 18, 07743 Jena
Telefon:	03641 9-325 624
Fax:	03641 9-399 925
E-Mail:	Datenschutzbeauftragter@med.uni-jena.de

8. Mir/Uns ist bekannt, dass wir bezogen auf die Verarbeitung der personenbezogenen Daten ein Beschwerderecht bei einer Datenschutz-Aufsichtsbehörde haben. Dies ist

für Thüringen

Thüringer Datenschutzaufsichtsbehörde

Thüringer Landesbeauftragter für den Datenschutz und die Informationsfreiheit (TLfDI)

Adresse: Postfach 900455, 99107 Erfurt

Telefon: 0361 57-311 29 00

Fax: 0361 57-311 29 04

E-Mail: poststelle@datenschutz.thueringen.de

9. Mir/uns ist bekannt, dass wir bezogen auf die Verarbeitung der personenbezogenen Daten ein Beschwerderecht bei der Bundesdatenschutzbeauftragte haben:

Bundesdatenschutzbeauftragte:

Die Bundesbeauftragte für den Datenschutz und die Informationsfreiheit

Adresse: Husarenstr. 30, 53117 Bonn

Telefon: 0228 997799-0

Fax: 0228 997799-550

E-Mail: poststelle@bfdi.bund.de

10. Mir/uns ist bekannt, dass wir ein Recht auf Auskunft über die verarbeiteten personenbezogenen Daten meines/unseres Kindes haben. Dieses Auskunftsrecht besteht gegenüber dem unter Punkt 7 genannten Verantwortlichen.
11. Mir/uns ist bekannt, dass ich/wir ein Recht auf unverzügliche Berichtigung (eingeschränkt) Übertragung und Löschung meiner/unserer personenbezogenen Daten habe.

PATIENT

Mit meiner/unserer Unterschrift erkläre/n ich mich/wir uns bereit, dass mein/unser Kind an der oben genannten klinischen Studie freiwillig teilnimmt.

Eine Kopie der Patienteninformation und -Einwilligung sowie die Versicherungsbedingungen habe/n ich/wir erhalten. Ein Exemplar verbleibt im Prüfzentrum.

Name des Patienten in Druckbuchstaben
(eigenhändig von einem Elternteil/eines Sorgeberechtigten einzutragen)

/ /

Datum
(eigenhändig vom Sorgeberechtigten einzutragen)

Unterschrift Sorgeberechtigter 1

/ /

Datum
(eigenhändig vom Sorgeberechtigten einzutragen)

Unterschrift Sorgeberechtigter 2

ARZT

Ich habe das Aufklärungsgespräch geführt und die Einwilligung des Patienten eingeholt.

Name der Prüfärztin / des Prüfarztes in Druckbuchstaben

/ /

Datum

Unterschrift der Prüfärztin / des Prüfarztes in Druckbuchstaben
