# Supplementary material for: Tonsillectomy versus tonsillotomy for recurrent acute tonsillitis in children and adults (TOTO): study protocol for a randomized non-inferiority trial
Source: Trials. 2021 Jul 22;22:479. doi: 10.1186/s13063-021-05434-y (PMC8296750; doi:10.1186/s13063-021-05434-y)
Supplement: Supplementary file 2 — Additional file 2. Toto consent 3–6 years. [file 13063_2021_5434_MOESM2_ESM.pdf]

INFORMATION FÜR PATIENTEN

(empfohlen für die Altersgruppe drei bis sechs Jahre)

Informationsblatt und Einwilligungserklärung zur Studie:

Tonsillektomie versus Tonsillotomie bei Kindern und Erwachsenen mit rezidivierender akuter Tonsillitis: Eine kontrollierte, randomisierte Nichtunterlegenheits-Studie

Kurztitel	Toto
Prüfplan-Nummer	UMG20775
DRKS Nummer	DRKS 00020283
Version	1.0 vom 17.04.2020

Name, Anschrift und Telefonnummer Ihres Prüfarztes:

INHALT

Warum werde ich befragt und wie hängt das mit meinen Halsschmerzen zusammen? 3

Was soll ich tun? 4

Was wird mit mir geschehen und welche Risiken können bestehen? 4

Kann ich meine Meinung ändern? 5

Wen kann ich fragen? 5

Einverständniserklärung 6

Liebe Patientin, lieber Patient,

Du hast immer wieder Halsschmerzen und musst deswegen oft zum Arzt. Deine Krankheit heißt Mandelentzündung (die Ärzte sprechen hier von einer Tonsillitis). Dabei sind Deine Mandeln krank. Um gesund zu werden, sollst Du an den Gaumenmandeln (kurz meist Mandeln genannt) operiert werden.

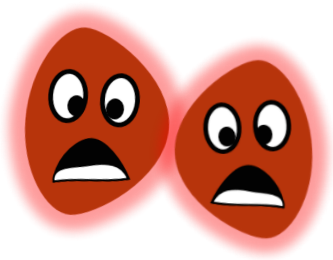

Das sind entzündete Gaumenmandeln, die Dir oft sehr große Schmerzen bereiten.

Warum werde ich befragt und wie hängt das mit meinen Halsschmerzen zusammen?

Wie du weißt, hast Du entzündete Mandeln und dadurch immer wieder Halsschmerzen. Du fühlst Dich gar nicht gut und hast sogar Fieber.

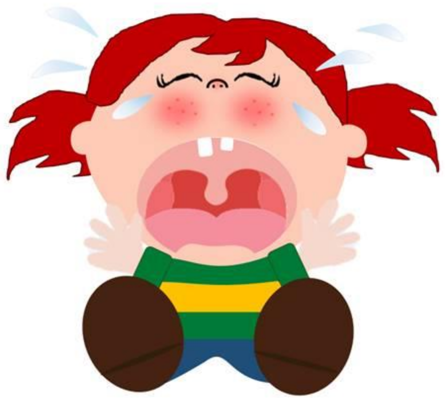

Schau mal! Das ist die kleine Nicki.
Nicki geht es gar nicht gut – so wie Dir, wenn Du Halsschmerzen und Fieber hast.

Hast Du diese Schmerzen öfter und stehen sie in Bezug zu der Mandelentzündung, werden die Mandeln, die in Deinem Gaumen sitzen, oft mit einer Operation herausgenommen. Der Arzt macht das in zwei verschiedenen Operationsverfahren. Entweder nimmt er Deine Gaumenmandeln ganz heraus oder nur einen Teil davon. Auch wenn die Ärzte beide Verfahren regelmäßig anwenden, weiß niemand, ob das eine Verfahren schlechter als das andere für Patienten mit wiederkehrender Mandelentzündung ist.

In vielen Ländern bemühen sich Forscher und Ärzte, Unterschiede zwischen den Operationsverfahren zu erkennen und für eine bessere Behandlung der Patienten zu

berücksichtigen. Dazu werden Klinische Studien gemacht. Gibt es verschiedene Operationsmöglichkeiten zu Auswahl, prüfen Ärzte hierbei welches Verfahren am besten ist.

Welche Operation auch bei Kindern am besten funktioniert, wollen die Ärzte mit Dir zusammen erforschen. Klingt eigentlich ganz spannend, oder? Du kannst dabei helfen, dass wir beide Operationsverfahren besser verstehen und dass es der kleinen Nicki bald wieder besser geht.

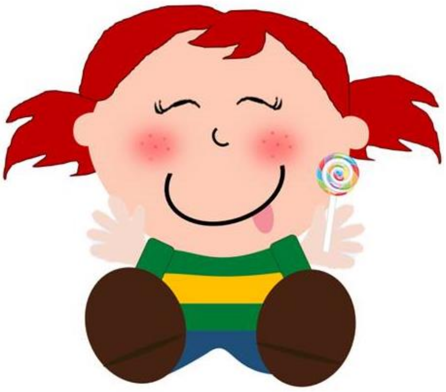

Was soll ich tun?

Wir möchten Dich nun fragen, ob du bei der Untersuchung mitmachen möchtest. Wir wollen ein Verfahren überprüfen, dass Dir vielleicht hilft, künftig weniger Halsschmerzen zu haben. Das Verfahren wurde schon bei vielen Kindern wie Dir ausprobiert und wirkt meistens gut. Damit wir noch weiteren Kindern und Patienten helfen können, so wie Nicki, müssen wir viel mehr über diese operativen Verfahren herausfinden.

Was wird mit mir geschehen und welche Risiken können bestehen?

Wenn du mitmachst, wird mit einem bestimmten Prinzip (vergleichbar einem Münzwurf) ein operatives Verfahren für Dich ausgewählt. Als mögliche Operationen können Deine Gaumenmandeln ganz oder nur ein Teil davon herausgenommen werden.

Jede Operation, so auch Deine Mandeloperation, kann mit bestimmten Risiken, wie Blutungen und Schmerzen verbunden sein. Diese Risiken bestehen auch woanders, wenn Du dich operieren lässt. Was genau bei der Operation passieren kann, wird Dir Dein Arzt noch einmal erklären.

Bei der Operation wirst Du tief und fest schlafen, so dass der Arzt – ohne dass Du während des Eingriffes Schmerzen hast - deine Gaumenmandeln herausnehmen kann. Nach der Operation wird es Dir wahrscheinlich kurzfristig nicht gut gehen, da Du durch das Herausnehmen der Mandeln oft auch Blutungen haben könntest. Dies sollte aber nur kurz dauern und langfristig sollten Deine Halsschmerzen weniger werden.

Um dies zu überprüfen wollen wir Dich und Deine Eltern regelmäßig (und zwar jede Woche über einen Zeitraum von zwei Jahren) fragen, ob Du Halsschmerzen hast und wenn ja, wie stark sie sind. Hierzu geben wir Deinen Eltern die Möglichkeit über ein Tagebuch oder das Internet oder eine App, Fragen zu deinen Halsschmerzen zu beantworten.

Zusätzlich werden wir Dich und Deine Eltern in regelmäßigen Abständen (und zwar alle sechs Monate über einen Zeitraum von zwei Jahren) telefonisch kontaktieren, um Dir Fragen zu Deinem Wohlbefinden zu stellen. Bei eventuellen Rückfragen möchte Dich Dein Studienzentrum (Prüfzentrum) vielleicht auch gerne zwischendurch einmal anrufen dürfen.

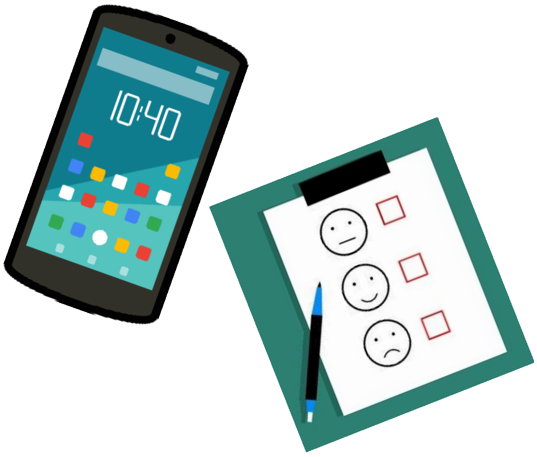

Deine Eltern sind die ganze Zeit bei dir. Wenn Dir irgendetwas, was der Arzt tut, nicht gefällt, kannst Du das jederzeit sagen.

Kann ich meine Meinung ändern?

Es ist nicht schlimm, wenn Du bei dieser Studie nicht mitmachen möchtest. Sag das einfach Deinen Eltern oder deinem Arzt.

Wen kann ich fragen?

Du kannst immer Deine Eltern oder den Arzt fragen. Denke nicht, dass Deine Fragen dumm sind. Wenn Du etwas wissen möchtest oder nicht verstehst, dann darfst Du uns Ärzte immer gern fragen.

Vielen Dank!

Vielen Dank, dass Du bei der Untersuchung mitmachst und hilfst, dass es Nicki und vielen anderen Kindern bald wieder besser geht. Ich bin gespannt, was wir zusammen herausfinden.

Einverständniserklärung

Toto:

Toto: Tonsillektomie versus Tonsillotomie bei Kindern und Erwachsenen mit rezidivierender akuter Tonsillitis: Eine kontrollierte, randomisierte Nichtunterlegenheits-Studie

Den folgenden Teil soll die Patientin / der Patient selbst ausfüllen. Bei Patienten unter 6 Jahren kann die Einwilligung auch mündlich erfolgen. Dies ist vom Prüfarzt entsprechend zu dokumentieren.

Wenn Du bei der Studie mitmachen möchtest, bitten wir Dich, die folgenden Fragen zu beantworten. Du sagst uns damit, dass Du an der Studie teilnehmen möchtest und weißt, dass dies freiwillig ist. Du kannst aber auch später zu jeder Zeit sagen, dass Du nicht mehr an der Studie teilnehmen möchtest. Du wirst dadurch auch keine Nachteile haben.

Hast du das Informationsblatt gelesen oder wurde es Dir vorgelesen?	Ja	Nein
Hast Du alles verstanden?	Ja	Nein
Hast du alle Fragen gestellt, die Du stellen wolltest?	Ja	Nein
Hat der Arzt alle Deine Fragen beantwortet?	Ja	Nein
Weißt Du, wie Du mit der Studie aufhören kannst, wenn Du das möchtest?	Ja	Nein
Möchtest Du teilnehmen?	Ja	Nein

PATIENT

Name des Patienten in Druckbuchstaben
(eigenhändig vom Patienten einzutragen)

/ /

Datum
(eigenhändig vom Patienten einzutragen)

Unterschrift des Patienten

ARZT

Ich habe das Aufklärungsgespräch geführt und die Einwilligung des Kindes eingeholt. Ich habe mich davon überzeugt, dass das Kind alles verstanden hat, keine weiteren Fragen mehr hat und die Teilnahme nicht ablehnt.

Name der Prüfärztin / des Prüfarztes in Druckbuchstaben

/ /

Datum

Unterschrift der Prüfärztin /
des Prüfarztes in Druckbuchstaben
