# Supplementary material for: Tonsillectomy versus tonsillotomy for recurrent acute tonsillitis in children and adults (TOTO): study protocol for a randomized non-inferiority trial
Source: Trials. 2021 Jul 22;22:479. doi: 10.1186/s13063-021-05434-y (PMC8296750; doi:10.1186/s13063-021-05434-y)
Supplement: Supplementary file 3 — Additional file 3. Toto consent 7–11 years. [file 13063_2021_5434_MOESM3_ESM.pdf]

INFORMATION FÜR PATIENTEN

(empfohlen für die Altersgruppe sieben bis elf Jahre)

Informationsblatt und Einwilligungserklärung zur Studie:

Tonsillektomie versus Tonsillotomie bei Kindern und Erwachsenen mit rezidivierender akuter Tonsillitis: Eine kontrollierte, randomisierte Nichtunterlegenheits-Studie

Kurztitel	Toto
Prüfplan-Nummer	UMG20775
DRKS Nummer	DRKS 00020283
Version	1.0 vom 17.04.2020

Name, Anschrift und Telefonnummer Ihres Prüfarztes:

INHALT

Informationen zur Studie 3

Warum wird die Studie durchgeführt? 3

Warum fragen wir Dich und wie hängt das mit Deinen Halsschmerzen zusammen? 3

Was wird bei der Studie gemacht und was geschieht mit mir? 4

Werde ich auf jeden Fall operiert? 4

Welche Risiken bestehen? 4

Muss ich bis zum Ende der Studie mitmachen? 5

…und wenn ich Fragen habe? 5

Einverständniserklärung 6

Liebe Patientin, lieber Patient,

wir möchten Dich fragen, ob Du bereit bist, bei der klinischen Prüfung (Studie) mitzumachen.

Studien wie diese dienen der Forschung in der Medizin. Sie sind notwendig, um genauer herauszufinden, wie gut Medikamente oder bestimmte medizinische Verfahren wirken und wie gut sie vertragen werden.

Ob Du bei der Studie mitmachen möchtest, kannst Du zusammen mit Deinen Eltern entscheiden. Keiner zwingt Dich zur Teilnahme. Du kannst auch einfach nein sagen. Wenn Du nicht an der Studie teilnehmen oder später aus ihr ausscheiden möchtest, entstehen Dir daraus keine Nachteile.

Du musst Dich nicht gleich entscheiden; lass Dir ruhig Zeit, um darüber nachzudenken. Diese Information soll Dir bei Deiner Entscheidung helfen, denn es ist wichtig, dass Du alles verstehst. Auch Deine Eltern haben von uns eine schriftliche Information bekommen. Sie werden sicher die meisten Fragen, die Du hast, beantworten können. Und Dein Arzt wird zu dieser Studie auch alles mit Dir besprechen.

Informationen zur Studie

Warum wird die Studie durchgeführt?

Warum fragen wir Dich und wie hängt das mit Deinen Halsschmerzen zusammen?

Du wirst wegen immer wiederkehrender Halsschmerzen von Deinen Ärzten behandelt. Oft hast Du auch Fieber, wenn Du Halsschmerzen hast. Wenn die Halsschmerzen im Zusammenhang mit Deinen Gaumenmandeln stehen, spricht man von einer Mandelentzündung.

Bei häufig wiederkehrenden Mandelentzündungen werden die Gaumenmandeln oft mit einer Operation herausgenommen.

Zur Operation an den Mandeln gibt es zwei Verfahren, die zur Anwendung kommen. In dem einen Verfahren werden die Gaumenmandeln vollständig – in dem anderen Verfahren nur teilweise entnommen. Auch wenn beide Verfahren regelmäßig zur Anwendung kommen, weiß niemand, ob das eine Verfahren schlechter als das andere ist.

In vielen Ländern bemühen sich Forscher und Ärzte, Unterschiede zwischen den Operationsverfahren zu erkennen und zu berücksichtigen. Dazu werden Studien gemacht. Bei Operationen prüfen Ärzte hierbei verschiedene Verfahren um Wissenslücken zu schließen oder beispielsweise die besten Verfahren zu finden, um besser helfen zu können.

Ob die unterschiedlichen Operationen auch bei Kindern funktionieren, wollen die Ärzte mit dir zusammen erforschen. Klingt eigentlich ganz spannend. Du kannst dabei helfen, dass wir hier ein besseres Verständnis der beiden Verfahren gewinnen können.

Was wird bei der Studie gemacht und was geschieht mit mir?

Bei Aufnahme in diese Studie fragen wir zunächst Dich und Deine Eltern, ob Du bereit bist mitzumachen. Hierbei stellen wir auch bestimmte Fragen zu Deiner Gesundheit und erheben bestimmte Daten (wie beispielsweise Dein Gewicht, Deine Größe, Dein Alter).

Wenn Du bereit bist bei der Studie mitzumachen wird mit einem bestimmten Prinzip (vergleichbar mit einem Münzwurf) ein operatives Verfahren für Dich ausgewählt. Als mögliche Operationen können vollständige oder nur teilweise Entnahme der Mandeln infrage kommen. Langfristig sollten nach der Operation Deine Halsschmerzen geringer werden.

Um dies zu überprüfen, wollen wir Dich und Deine Eltern regelmäßig (und zwar wöchentlich über einen Zeitraum von zwei Jahren) fragen, ob Du Halsschmerzen hast und wenn ja, wie stark sie sind. Hierzu geben wir Deinen Eltern die Möglichkeit über ein Tagebuch oder das Internet oder eine App, Fragen zu Deinen Halsschmerzen zu beantworten.

Zusätzlich werden wir Dich und Deine Eltern in regelmäßigen Abständen (und zwar alle sechs Monate über einen Zeitraum von zwei Jahren) telefonisch kontaktieren, um Dir Fragen zu Deinem Wohlbefinden zu stellen. Bei eventuellen Rückfragen möchte Dich Dein Studienzentrum (Prüfzentrum) vielleicht auch gerne zwischendurch einmal anrufen dürfen.

Deine Eltern sind die ganze Zeit bei Dir. Wenn Du irgendetwas, was der Arzt tut, nicht verstehst oder wenn es Dir nicht gefällt, kannst Du das immer sagen.

Werde ich auf jeden Fall operiert?

Unabhängig von der Studie hat der Arzt Deinen Eltern empfohlen, dass Du operiert wirst. Die Studie nimmt lediglich Einfluss auf die Auswahl der Art der Operation (vollständige oder teilweise Entnahme der Gaumenmandeln). Hierüber wird in der Studie zufällig entschieden. Aber egal welches Verfahren bei Dir verwendet werden würde, handelt es sich um ein Verfahren, bei dem die Ärzte bereits viele Erfahrungen haben.

Welche Risiken bestehen?

Jede Operation, so auch eine Operation an den Mandeln, kann Dir helfen, Dir gleichzeitig aber auch Schmerzen und Unwohlsein bereiten (Ärzte sprechen hier von bestimmten Risiken). Dies hat nichts mit Deiner Teilnahme an dieser Studie zu tun. Diese Risiken bestehen auch, wenn eine Mandeloperation außerhalb dieser Studie vorgenommen wird.

Wie Du weißt wird die Operation auf zwei verschiedene Arten durchgeführt. Im Folgenden sind zu beiden Operationen die Risiken aufgelistet.

Bei einer teilweisen Entnahme der Gaumenmandeln (Ärzte sprechen hier von einer Tonsillotomie) kannst Du im Hals nachbluten oder bluten. Deine Stimme kann sich ein wenig verändern (durch die Nase sprechen). Du kannst vielleicht Beschwerden beim Schlucken haben oder Beschwerden an Zahn-, Zungen- und Deiner Schleimhaut sowie Überschlucken in die Nase.

Bei einer kompletten Entnahme der Gaumenmandeln (Ärzte sprechen hier von Tonsillektomie) kannst Du im Hals nachbluten bis zu 2 Wochen nach der Operation und Dein Hals könnte auch danach immer noch mal wieder bluten mit möglicherweise tödlichem Ausgang.

Deine Stimme kann sich ein wenig verändern, durch die Nase sprechen. Du kannst vielleicht Beschwerden beim Schlucken haben oder Beschwerden an Deiner Zahn-, Zungen- und Schleimhaut sowie Überschlucken in die Nase. Bezüglich der Operation und den damit verbundenen speziellen Risiken wirst DU separat informiert und aufgeklärt.

Muss ich bis zum Ende der Studie mitmachen?

Wenn Du Dich dazu entschließt, dass Du bei dieser Untersuchung nicht mehr mitmachen möchtest, sagst Du das einfach Deinen Eltern. Du kannst es auch Deinem Arzt sagen. Solltest Du aufhören, wirst Du außerhalb der Studie weiterhin gut behandelt.

...und wenn ich Fragen habe?

Wenn Du Fragen hast zu dem, was mit Dir geschieht, kannst Du Deine Eltern oder die Ärzte fragen. Sie werden alle Deine Fragen beantworten. Denke nicht, dass Deine Fragen dumm sind. Wenn Du etwas wissen möchtest oder nicht verstehst, dann darfst Du uns Ärzte immer gerne fragen.

Einverständniserklärung

Toto: Tonsillektomie versus Tonsillotomie bei Kindern und Erwachsenen mit rezidivierender akuter Tonsillitis: Eine kontrollierte, randomisierte Nichtunterlegenheits-Studie

Wenn Du Fragen hast zu dem, was mit Dir geschieht, kannst Du Deine Eltern oder die Ärzte fragen. Sie werden alle Deine Fragen beantworten. Denke nicht, dass Deine Fragen dumm sind. Wenn Du etwas wissen möchtest oder nicht verstehst, dann darfst Du uns Ärzte immer gerne fragen.

Ich möchte an dieser Studie teilnehmen.

PATIENT

Name des Patienten in Druckbuchstaben
(eigenhändig vom Patienten einzutragen)

/ /

Datum
(eigenhändig vom Patienten einzutragen)

Unterschrift des Patienten

ARZT

Ich habe das Aufklärungsgespräch geführt und die Einwilligung des Kindes eingeholt. Ich habe mich davon überzeugt, dass das Kind alles verstanden hat, keine weiteren Fragen mehr hat und die Teilnahme nicht ablehnt.

Name der Prüfärztin / des Prüfarztes in Druckbuchstaben

/ /

Datum

Unterschrift der Prüfärztin / des Prüfarztes in Druckbuchstaben
