# Supplementary material for: Tonsillectomy versus tonsillotomy for recurrent acute tonsillitis in children and adults (TOTO): study protocol for a randomized non-inferiority trial
Source: Trials. 2021 Jul 22;22:479. doi: 10.1186/s13063-021-05434-y (PMC8296750; doi:10.1186/s13063-021-05434-y)
Supplement: Supplementary file 4 — Additional file 4. Toto consent 12–15 years. [file 13063_2021_5434_MOESM4_ESM.pdf]

INFORMATION FÜR PATIENTEN

(empfohlen für die Altersgruppe zwölf bis fünfzehn Jahre)

Informationsblatt und Einwilligungserklärung zur Studie:

Tonsillektomie versus Tonsillotomie bei Kindern und Erwachsenen mit rezidivierender akuter Tonsillitis: Eine kontrollierte, randomisierte Nichtunterlegenheits-Studie

Kurztitel	Toto
Prüfplan-Nummer	UMG20775
DRKS Nummer	DRKS 00020283
Version	1.0 vom 17.04.2020

Name, Anschrift und Telefonnummer Ihres Prüfarztes:

INHALT

Informationen zum Studienablauf 4
Allgemeine Information zur Studie 4
Warum wird die Studie durchgeführt? 4
Operative Verfahren bei Mandelentzündungen 4
Kann ich entscheiden, welches Verfahren bei mir zur Anwendung kommt? 5
Ziel der Studie 5
Was ist der Ablauf der Studie und was muss ich bei einer Teilnahme wissen? 5
Voruntersuchung 5
Dauer der Studie 5
Ablauf der Studie und Untersuchungen im Laufe der Studie 5
Zeitlicher Ablauf der Studie 6
Was musst du bei einer Studienteilnahme beachten? 6
Was bringt es mir, wenn ich an der Studie teilnehme? 6
Welche Risiken bestehen? 7
Behandlungsmöglichkeiten außerhalb der Studie 7
Wer darf bei dieser Studie nicht teilnehmen? 7
Was kostet mich die Studie? Erhalte ich Geld zurück? 7
Bin ich während der klinischen Prüfung versichert? 7
Mitteilung neuer Erkenntnisse während der klinischen Prüfung 8
Beendigung der klinischen Prüfung 8
Datenschutz 8
Bei weiteren Fragen 8
Persönliche Notizen 9
Einverständniserklärung 10

Liebe Patientin, lieber Patient,

im Folgenden erhältst Du eine kleine Zusammenfassung der nachfolgenden Patienteninformation. Diese soll Dir lediglich einen Überblick verschaffen; ersetzt jedoch nicht die Inhalte der ausführlichen Patienteninformation. Wir bitten Dich daher, auch die Patienteninformation vollständig durchzulesen. Ob Du bei der Studie mitmachen möchtest, kannst Du zusammen mit Deinen Eltern entscheiden. Keiner zwingt Dich zur Teilnahme. Du kannst auch einfach nein sagen. Wenn Du nicht an der Studie teilnehmen oder später aus ihr ausscheiden möchtest, entstehen Dir daraus keine Nachteile.

- Mit der nachfolgenden Patienteninformation klären wir Dich über die Teilnahme an der klinischen Studie „TOTO" auf. Deine Teilnahme ist freiwillig und setzt Dein Einverständnis voraus.
- Die vorliegende Studie beschäftigt sich mit Mandelentzündungen.
- Die Studie wird von der Universitätsklinik Jena in Zusammenarbeit mit der Deutschen Gesellschaft Hals Nasen Ohren Heilkunde und dem Berufsverband Hals Nasen Ohren Heilkunde sowie dem Studienzentrum der Universitätsmedizin Göttingen organisiert.
- Wir möchten mit Hilfe dieser Studie untersuchen, wie sich zwei unterschiedliche Operationsverfahren auf Deine Erkrankung auswirkt.
- Einem Teil der Patienten werden die Gaumenmandeln **vollständig entfernt**. Einem anderen Teil werden die Gaumenmandeln **teilweise entfernt**.
- Welches operative Verfahren bei Dir zum Einsatz kommt, wird im Rahmen der Studienzuteilung nach zuvor festgelegten Zufallsverfahren, vergleichbar mit dem Werfen einer Münze, entschieden.
- Die Studie dauert **zwei Jahre**.
- Wenn Du nicht an der Studie teilnehmen möchtest, wird Dein behandelnder Arzt eine Therapie für Dich wählen, die Du auch bei einer Studienteilnahme bekommen hättest. Du kannst die **Teilnahme an der Studie** jederzeit auch während der Studie **beenden**, ohne dass Dir dadurch Nachteile entstehen.
- Du kannst NICHT an der Studie teilnehmen, wenn Du gleichzeitig **an anderen klinischen Studien teilnimmst** oder innerhalb von 4 Wochen vor Studienbeginn teilgenommen hast.
- Ebenso nicht teilnehmen können **schwangere** oder **stillende Mädchen**.
- Durch die Teilnahme an dieser klinischen Studie entstehen Dir und Deinen Eltern **keine zusätzlichen Kosten**.
- Alle Patienten die an der Studie teilnehmen sind **versichert**.
- Im Rahmen der Studie werden **persönliche Daten** von Dir erhoben und **gespeichert**. Dies erfolgt in **pseudonymisierter Form.**

Liebe Patientin, lieber Patient,

wir möchten Dich fragen, ob Du bereit bist, bei der klinischen Prüfung mitzumachen, die wir hier beschreiben (sie wird ab jetzt „klinische Studie" genannt).

Solche Studien dienen der Forschung in der Medizin. Sie sind notwendig, um genauer herauszufinden, ob und wie gut neue Medikamente oder wie bestimmte medizinische Verfahren wirken und wie gut sie vertragen werden.

Ob Du bei der Studie mitmachen möchtest, kannst Du zusammen mit Deinen Eltern selbst entscheiden. Deine Teilnahme ist, auch wenn Deine Eltern zustimmen, freiwillig; keiner zwingt Dich. Wenn Du nicht an der Studie teilnehmen oder später aus ihr ausscheiden möchtest, entstehen Dir daraus keine Nachteile. Ob Du mitmachen willst, musst Du nicht gleich entscheiden; lass Dir ruhig einige Tage Zeit, um darüber nachzudenken. Diese Information soll Dir bei Deiner Entscheidung helfen, denn es ist wichtig, dass Du alles verstehst. Auch Deine Eltern haben von uns eine schriftliche Information bekommen. Wenn Ihr alles gelesen habt, werdet Ihr Euch darüber unterhalten. Deine Eltern werden sicher die meisten Fragen, die Du hast, beantworten können. Und Dein Arzt wird wegen dieser Studie auch alles mit Dir besprechen. Er wird genügend Zeit haben, Deine Fragen zu beantworten.

Informationen zum Studienablauf

Allgemeine Information zur Studie

Die klinische Studie, die wir Dir hier vorstellen wurde von einer Ethikkommission erlaubt. Insgesamt sollen 454 Patienten unterschiedlichen Alters an mehreren Orten in Deutschland mitmachen. Die Studie wird von der Universitätsmedizin Jena in Zusammenarbeit mit dem Studienzentrum Göttingen organisiert. Bezahlt wir die Studie durch den gemeinsamen Bundesaus-schuss. Die operativen Verfahren, die in dieser Studie betrachtet werden, werden von der Krankenkasse vergütet.

Warum wird die Studie durchgeführt?

Operative Verfahren bei Mandelentzündungen

Bei chronischen Mandelentzündungen finden derzeit zwei operative Verfahren standardmäßig in der Medizin Anwendung. Bei dem Einen handelt es sich um eine teilweise Entfernung der Gaumenmandeln (Tonsillotomie), bei dem Anderen um eine vollständige Entfernung der Gaumenmandeln (Tonsillektomie). Bislang konnte noch nicht geklärt werden, ob bei Patientinnen und Patienten mit immer wiederkehrenden akuten Mandelentzündungen, bei der der behandelnde Arzt einen operativen Eingriff empfehlen würde, eine Tonsillotomie gegenüber einer Tonsillektomie nicht unterlegen ist. Mit der Durchführung dieser Studie soll diese Fragestellung geklärt werden.

Kann ich entscheiden, welches Verfahren bei mir zur Anwendung kommt?

Im Rahmen dieser Studie wird die Tonsillotomie mit der Tonsillektomie verglichen, um Wirkungen und Nebenwirkungen der Verfahren besser beurteilen zu können. Beide Verfahren sind für die Therapie bei wiederkehrenden akuten Mandelentzündungen etabliert und zugelassen. Deshalb werden alle Patienten, die an der Studie teilnehmen, in zwei Gruppen eingeteilt. Die eine Gruppe erhält eine Tonsillotomie, die andere Gruppe eine Tonsillektomie.

Zu welcher Gruppe Du im Falle Deiner Teilnahme gehörst, wird nach Zufallsprinzip entschieden; vergleichbar mit dem Werfen einer Münze. Die Wahrscheinlichkeit, dass Du eine Tonsillotomie erhältst, beträgt 50%. Deine Eltern und der Arzt wissen aber in welcher Gruppe Du bist.

Ziel der Studie

Das Hauptziel der Studie ist es, zu zeigen, dass die Tonsillotomie nicht weniger gut hilft, als die Tonsillektomie.

Weitere Ziele sind u.a.:

- Die systematische Erfassung von Nebenwirkungen und Anzahl der Tage an denen Halsschmerzen nach der Operation auftreten

Was ist der Ablauf der Studie und was muss ich bei einer Teilnahme wissen?

Voruntersuchung

Wenn Du bei der Studie mitmachst, wird zuerst die Vorgeschichte Deiner Krankheit abgefragt und Du wirst umfassend ärztlich untersucht. Dazu gehören Fragen zu Deiner Krankheitsgeschichte und auch medizinische Untersuchungen (bspw. Gewicht, Größe). Ob Du an der klinischen Studie mitmachen kannst, hängt von den Ergebnissen dieser Voruntersuchung ab.

Dauer der Studie

Die Operation und die danach folgende Behandlung dauert nur wenige Tage. Nach der Operation wollen wir jedoch über einen Zeitraum von zwei Jahren wissen, ob Du noch weiterhin regelmäßig Halsschmerzen hast und falls ja, wie stark diese Halsschmerzen sind.

Ablauf der Studie und Untersuchungen im Laufe der Studie

Wenn Du der Studienteilnahme zugestimmt hast und die Eingangsuntersuchungen durchgeführt wurden, erfolgt die zufällige Zuteilung in einen der beiden Behandlungsgruppen (wie auf der vorherigen Seite beschrieben).

- In einer Operation werden Dir Deine Gaumenmandeln teilweise oder vollständig entfernt
- Über einen Zeitraum von 24 Monaten werden wir Dir wöchentlich Fragen stellen, die,
    - Die Häufigkeit und Schwere der Halsschmerzen betreffen.
    - Fragen zur Lebensqualität beinhalten.

Hierzu werden wir Dir und/oder Deinen Eltern, bzw. Erziehungsberechtigten die Möglichkeit geben, die Fragen mithilfe einer App, eines Internetzugangs oder eines Tagebuchs zu beantworten.

Zusätzlich werden wir Dich und Deine Eltern in regelmäßigen Abständen (und zwar alle sechs Monate über einen Zeitraum von zwei Jahren) telefonisch kontaktieren, um Dir Fragen zu Deinem Wohlbefinden zu stellen. Bei eventuellen Rückfragen möchte Dich Dein Studienzentrum (Prüfzentrum) vielleicht auch gerne zwischendurch einmal anrufen dürfen.

Zeitlicher Ablauf der Studie

Bezeichnung/Monat	**Voruntersuchungen**	**Operation**[a]	**wöchentliche Patientendatenerhebungen (bis Monat 24)**[b]	**Nachunter-suchungen Monat**[b] **6, 12, 18, 24**
Ein-Ausschlusskriterien	X			
Patientendaten	X			
Einwilligungserklärung	X			
Randomisierung	X			
OP Daten		X		
Blutungen / UEs*		X ----------	----------X----------	----------X
Anzahl Tage Halsschmerzen / NRS	X		X	X
STAR (Wenn Halschmerzen vorliegen)**			X	X
TAHSI**				X
TOI**				X
SF-12**				X

[a] = am Zentrum

[b] = Datenerhebung durch Patient [ggf. Weiterleitung Daten (bspw. Tagebuch)]

* Erfassung bei Anfall (bspw. Blutungen am behandelnden Zentrum)

** Bei Weiterleitung der Daten: Arbeitsanfall am Zentrum und zentrale Datenerfassung

Was musst du bei einer Studienteilnahme beachten?

- Da Du nach der Operation nicht mehr in der Klinik und in der Regel Deinen HNO Arzt nicht regelmäßig besuchen wirst, ist es wichtig, dass Du die Fragen nach Deinen Hallschmerzen wöchentlich beantwortest.
- Wenn Deinem, in dieser Patienteninformation genannten, behandelnden Arzt auffällt, dass Du die Fragen nicht regelmäßig beantwortest, kann es sein, dass der Arzt bei Dir nachfragen und Dich bitten wird, die Fragen regelmäßig zu erfassen.
- Teile Deinen Eltern/Deinem Sorgeberechtigten/dem Studienpersonal alle Erkrankungen und Verletzungen einschließlich eine Verschlechterung deines Gesundheitszustands, die während der Studie bei dir auftreten mit.

Was bringt es mir, wenn ich an der Studie teilnehme?

Wenn Du operiert wirst, wirst Du längerfristig wahrscheinlich weniger Halsschmerzen haben. Wenn die Gaumenmandeln entfernt sind, können sich diese nicht mehr entzünden. Das Gewebe um die Mandeln, kann sich jedoch entzünden. Der Arzt würde Deinen Eltern, bzw. Sorgeberechtigten jedoch – unabhängig von der Studienteilnahme – empfehlen, dass Du operiert wirst. Du kannst jedoch einen anderen Arzt fragen, ob er zu der gleichen Einschätzung kommen würde.

Welche Risiken bestehen?

Jede Operation, so auch eine Mandeloperation, ist mit bestimmten Risiken versehen. Dies hat nichts mit Deiner Teilnahme an der Studie zu tun. Diese Risiken bestehen auch, wenn eine Mandeloperation außerhalb der Studie vorgenommen wird. Durch die Studienteilnahme ergibt sich also kein zusätzliches Risiko.

Bei einer teilweisen Entfernung der Gaumenmandeln (Ärzte sprechen hier von einer Tonsillotomie) können Nachblutung, Blutung, Sprachklangänderung (z.B. offenes Näseln), Schluckbeschwerden, Zahn-, Zungen-, Schleimhautschädigung und Überschlucken in die Nase als Risiken auftreten.

Bei einer kompletten Entfernung der Gaumenmandeln (Ärzte sprechen hier von Tonsillektomie) können Nachblutung (auch bis zu 14 Tage nach Operation) mit möglicherweise tödlichem Ausgang, Blutungen, Sprachklangänderung (z.B. offenes Näseln), Schluckbeschwerden, Zahn, Zungen-, Schleimhautschädigung und Überschlucken in die Nase als Risiken auftreten.

Bezüglich der Operation und den damit verbundenen speziellen Risiken wirst Du separat informiert und aufgeklärt.

Behandlungsmöglichkeiten außerhalb der Studie

Wenn Du nicht an der Studie teilnehmen möchtest, stehen zur Behandlung Deiner Erkrankung auch die folgenden Möglichkeiten zur Verfügung: Gabe von Medikamenten (Antibiotika) zur Bekämpfung des Infekts. Da aber bei Dir bislang bei früheren Entzündungen dieses Vorgehen gewählt wurde, ohne dass eine dauerhafte Besserung eingetreten ist, würde Dein Arzt – entsprechend den aktuellen medizinischen Leitlinien – eine Operation empfehlen.

Wer darf bei dieser Studie nicht teilnehmen?

Du darfst nicht teilnehmen, wenn Du gleichzeitig an anderen Studien teilnimmst oder vor kurzem teilgenommen hast.

Schwangere und stillende Mädchen dürfen an der klinischen Studie ebenfalls nicht teilnehmen.

Was kostet mich die Studie? Erhalte ich Geld zurück?

Durch Deine Teilnahme an dieser Studie entstehen für Dich und Deine Eltern/Deinen Sorgeberechtigen **keine** zusätzlichen Kosten. Fahrtkosten zum Studienzentrum werden nicht erstattet.

Bin ich während der klinischen Prüfung versichert?

Du bist während der Studie gegen eventuelle Gesundheitsschäden durch Deine Studienteilnahme versichert. Nähere Informationen dazu haben Deine Eltern erhalten. Das betrifft auch die Frage, was geschehen muss, wenn Du den Verdacht hast, dass die Studie bei Dir einen Gesundheitsschaden verursacht haben könnte.

Mitteilung neuer Erkenntnisse während der klinischen Prüfung

Wenn es neue Ergebnisse über die Operationsmethoden gibt, die für diese klinische Studie wichtig sind, sagen die Ärzte Dir das. Du kannst dann überlegen, ob Du weiter bei der Studie mitmachen möchtest.

Beendigung der klinischen Prüfung

Du kannst jederzeit, auch ohne Angabe von Gründen, Deine Teilnahme an der Studie beenden, ohne dass Dir dadurch irgendwelche Nachteile bei Deiner medizinischen Behandlung entstehen.

Unter gewissen Umständen ist es aber auch möglich, dass Deine Teilnahme aus anderen Gründen beendet werden muss. Das kann zum Beispiel sein, wenn:

- es für Dich gesundheitlich nicht gut ist, weiter mitzumachen;
- die gesamte klinische Studie abgebrochen wird.

Datenschutz

Während der Studie werden Angaben über Dich und Deine Krankheit aufgeschrieben und elektronisch gespeichert. Die für die Studie wichtigen Daten werden zusätzlich „pseudonymisiert" gespeichert, so dass nicht so einfach zu erkennen ist, von wem sie stammen. Die Ergebnisse werden ausgewertet und gegebenenfalls weitergegeben.

Pseudonymisiert bedeutet, dass keine Angaben von Namen oder Anfangsbuchstaben der Namen verwendet werden, sondern nur ein Nummern- und/oder Buchstabencode.

Die Daten sind gegen unbefugten Zugriff gesichert. Eine Entschlüsselung erfolgt nur unter den vom Gesetz vorgeschriebenen Voraussetzungen oder wenn die Behandlung von der vorher zugelosten Behandlung abhängt.

Nähere Informationen dazu haben Deine Eltern erhalten.

Bei weiteren Fragen

Beratungsgespräche an dem behandelnden Studienzentrum (Prüfzentrum)

Du kannst bei jeder Gelegenheit in Deinem Prüfzentrum fragen. Die Adresse steht auf der Seite 1.

Du erhältst eine Kopie dieser Patienteninformation und der unterschriebenen Einverständniserklärung.

Unabhängig von Deiner Entscheidung über eine Teilnahme an der Studie, wünschen wir Dir für Deinen weiteren Behandlungsverlauf **alles Gute**.

Falls Du keine weiteren Fragen hast und an der Studie teilnehmen möchtest, möchten wir Dich bitten, die beiliegende Einverständniserklärung zu unterschreiben.

Persönliche Notizen

Einverständniserklärung

Toto: Tonsillektomie versus Tonsillotomie bei Kindern und Erwachsenen mit rezidivierender akuter Tonsillitis: Eine kontrollierte, randomisierte Nichtunterlegenheits-Studie

Prüfzentrum *(Stempel)*

Prüfarzt: ______________________________

Name: ______________________________

Telefon: ______________________________

Voraussetzung für Deine Teilnahme ist, dass Du einverstanden bist. Wenn ja, bitten wir Dich, auf diesem Blatt zu unterschreiben. Du bestätigst uns damit, dass Du an der Studie teilnehmen möchtest und weißt, dass dies freiwillig ist.

Möglichkeit zur Dokumentation zusätzlicher Fragen zum Aufklärungsgespräch:

____

____

____

Ich hatte ausreichend Zeit, mich zu entscheiden.

Ich weiß, dass ich auch später zu jeder Zeit sagen kann, dass ich nicht mehr an der Studie teilnehmen möchte. Ich werde deshalb auch keine Nachteile für meine medizinische Behandlung haben.

PATIENT

Mit meiner Unterschrift erkläre ich mit bereit, an der oben genannten Studie freiwillig teilzunehmen.

Eine Kopie der Patienteninformation und -Einwilligung habe ich erhalten. Ein Exemplar verbleibt im Prüfzentrum.

Name des Patienten in Druckbuchstaben
(eigenhändig vom Patienten einzutragen)

/ /

Datum
(eigenhändig vom Patienten einzutragen)

Unterschrift des Patienten

ARZT

Ich habe das Aufklärungsgespräch geführt und die Einwilligung des Kindes eingeholt. Ich habe mich davon überzeugt, dass der Jugendliche alles verstanden hat, keine weiteren Fragen mehr hat und die Teilnahme nicht ablehnt.

Name der Prüfärztin / des Prüfarztes in Druckbuchstaben

/ /

Datum

Unterschrift der Prüfärztin /
des Prüfarztes in Druckbuchstaben
