# Supplementary material for: Tonsillectomy versus tonsillotomy for recurrent acute tonsillitis in children and adults (TOTO): study protocol for a randomized non-inferiority trial
Source: Trials. 2021 Jul 22;22:479. doi: 10.1186/s13063-021-05434-y (PMC8296750; doi:10.1186/s13063-021-05434-y)
Supplement: Supplementary file 5 — Additional file 5. Toto consent 16+ years. [file 13063_2021_5434_MOESM5_ESM.pdf]

INFORMATION FÜR PATIENTEN

Informationsblatt und Einwilligungserklärung zur Studie:

Tonsillektomie versus Tonsillotomie bei Kindern und Erwachsenen mit rezidivierender akuter Tonsillitis: Eine kontrollierte, randomisierte Nichtunterlegenheits-Studie

Kurztitel	Toto
Prüfplan-Nummer	UMG20775
DRKS Nummer	DRKS 00020283
Version	1.0 vom 17.04.2020

Name, Anschrift und Telefonnummer Ihres Prüfarztes:

Inhalt der Patienteninformation

INFORMATIONEN ZUM STUDIENABLAUF ... **5**
Organisation und Verantwortlichkeit ... 5
Kontaktdaten Prüfzentrum ... 6
Warum wird die Studie durchgeführt? ... 6
Zielsetzung der Studie ... 7
Wie ist der Ablauf der Studie und was muss ich bei einer Teilnahme wissen? ... 7
Welche Risiken bestehen? ... 9
Behandlungsmöglichkeiten außerhalb der Studie ... 10
Wer darf bei dieser Studie nicht teilnehmen? ... 10
Was kostet mich die Studie? Erhalte ich Geld zurück? ... 10
Bin ich während der klinischen Studie versichert? ... 10
Mitteilung neuer Erkenntnisse während der klinischen Studie ... 11
Öffentliche Registrierung der Studie und Veröffentlichungen ... 11
Beendigung der klinischen Studie ... 11
Beendigung der klinischen Studie ... 11
Datenschutz ... 12
Für weitere Fragen ... 12
Persönliche Notizen ... 13
EINVERSTÄNDNISERKLÄRUNG ... **14**
DATENSCHUTZ ... **16**

Sehr geehrte Patientin, sehr geehrter Patient,

im Folgenden erhalten Sie einen Überblick über die Inhalte der nachfolgenden Patienteninformation. Dieser soll einer ersten Orientierung dienen, kann jedoch nicht die Inhalte der ausführlichen Patienteninformation ersetzen. Wir bitten Sie daher, die Patienteninformation vollständig und gewissenhaft durchzulesen.

- Mit der nachfolgenden Patienteninformation klären wir Sie über die Teilnahme an der klinischen Studie „TOTO“ auf. Ihre Teilnahme ist freiwillig und setzt Ihr schriftliches Einverständnis voraus.
- Die vorliegende Studie beschäftigt sich mit Mandelentzündungen.
- Die Studie wird von der Universitätsklinik Jena in Zusammenarbeit mit der Deutschen Gesellschaft HNO und dem Berufsverband HNO sowie dem Studienzentrum der Universitätsmedizin Göttingen organisiert.
- Wir möchten mit Hilfe dieser Studie untersuchen, wie sich zwei unterschiedliche Operationsverfahren auf Ihre Erkrankung auswirkt.
- Einem Teil der Patienten werden **die Gaumenmandeln vollständig entfernt**. Einem anderen Teil werden **die Gaumenmandeln teilweise entfernt.**
- Welches operative Verfahren zum Einsatz kommt, wird im Rahmen der Studienzuteilung nach zuvor festgelegten Zufallsverfahren, vergleichbar mit dem Werfen einer Münze, entschieden.
- Die **Studie dauert** zwei Jahre.
- Wenn Sie nicht an der Studie teilnehmen möchten, wird Ihr behandelnder Arzt eine Therapie für Sie wählen, die Sie auch bei einer Studienteilnahme bekommen hätten. Sie können die **Teilnahme an der Studie jederzeit** auch während der Studie **beenden**, ohne dass Ihnen dadurch Nachteile entstehen.
- Sie können NICHT an der Studie teilnehmen, sollten Sie gleichzeitig **an anderen klinischen Studien teilnehmen** oder innerhalb von 4 Wochen vor Studienbeginn teilgenommen haben.
- Ebenso nicht teilnehmen können **schwangere** oder **stillende Frauen.**
- Durch die Teilnahme an dieser klinischen Studie entstehen Ihnen gegenüber der üblichen Therapie **keine zusätzlichen Kosten.**

- Alle Patienten die an der Studie teilnehmen sind **versichert.**

- Bei Rücktritt von der Studie können auf Wunsch bereits gewonnene Daten vernichtet werden. Sie können sich bei der Rücknahme der Einwilligung entscheiden, ob Sie mit der Auswertung des Materials bzw. Ihrer Studiendaten einverstanden sind oder nicht. Sollten Sie zu einem späteren Zeitpunkt Ihre Entscheidung ändern wollen, setzen Sie sich bitte mit dem Studienarzt in Verbindung.

  Im Rahmen der Studie werden **persönliche Daten** von Ihnen erhoben und **gespeichert**. Dies erfolgt in **pseudonymisierter Form.** Eine Weitergabe erfolgt nur in anonymisierter Form. Pseudonymisiwerung bedeutet, dass Ihnen im Rahmen der Studie eine Nummer als Studienteilnehmer zugewiesen wird, die einen Rückschluss auf Ihre Identität ausschließt oder wesentlich erschwert. Die Zuordnung zwischen Pseudonymisierungsnummer und Ihrem Namen kann nur an dem Sie behandelnden Studienzentrum (Prüfzentrum) erfolgen. Bei einer Anonymisierung wäre auch diese Zuordnung nicht mehr möglich.

Sehr geehrte Patientin, sehr geehrter Patient,

Ihr Einverständnis zur Teilnahme an einer klinischen Studie

wir möchten Sie fragen, ob Sie bereit sind, an der nachfolgend beschriebenen klinischen Studie teilzunehmen.

Solche Studien dienen der Forschung in der Medizin. Sie sind notwendig, um genauer herauszufinden, ob und wie gut neue Medikamente wirken oder wie bestimmte medizinische Verfahren wirken und wie gut sie vertragen werden.

Ihre Teilnahme an dieser klinischen Studie ist freiwillig. Sie werden in diese Studie also nur dann einbezogen, wenn Sie dazu schriftlich Ihre Einwilligung erklären. Sofern Sie nicht an der klinischen Studie teilnehmen oder später aus ihr Ausscheiden möchten, entstehen Ihnen daraus keine Nachteile.

Sie wurden bereits auf die geplante Studie angesprochen. Der nachfolgende Text soll Ihnen die Ziele und den Ablauf erläutern. Anschließend wird ein behandelnder Studienarzt (Prüfarzt) das Aufklärungsgespräch mit Ihnen führen. Bitte zögern Sie nicht, alle Punkte anzusprechen, die Ihnen unklar sind. Sie haben danach ausreichend Bedenkzeit um mit Ihren Angehörigen über die Studie zu sprechen und über Ihre Teilnahme zu entscheiden.

Informationen zum Studienablauf

Organisation und Verantwortlichkeit

Organisation und Verantwortlichkeit

Diese klinische Studie wird multizentrisch in ca. 20 Zentren in Deutschland durchgeführt; es sollen insgesamt 554 Patienten daran teilnehmen. Die Studie wird von der Universitätsmedizin Jena in Zusammenarbeit mit dem Studienzentrum Göttingen organisiert. Bezahlt wird die Studie durch den gemeinsamen Bundesausschuss. Die operativen Verfahren, die in dieser Studie betrachtet werden, werden von der Krankenkasse vergütet.

Die Studie wurde von der zuständigen Ethikkommission zustimmend bewertet.

Kontaktdaten Prüfzentrum

Kontaktdaten
Prüfzentrum

**Prüfzentrum** (Stempel):	**Leiter/in der klinischen Studie:**
	Herr Prof. Guntinas-Lichius<br><br>HNO-Klinik<br>Universitätsklinikum Jena<br>Am Klinikum 1<br>07747 Jena<br><br>Telefon: 03641-9-329301<br>E-Mail:<br>Orlando.Guntinas@med.uni-jena.de

Warum wird die Studie durchgeführt?

Warum wird die Studie durchgeführt

Operative Verfahren bei Mandelentzündungen

Zur Entfernung der Gaumenmandeln finden derzeit zwei operative Verfahren standardmäßig in der Medizin Anwendung. Bei dem Einen handelt es sich um eine teilweise Entfernung der Gaumenmandeln (Tonsillotomie), bei dem Anderen um eine vollständige Entfernung der Gaumenmandeln (Tonsillektomie). Die teilweise Entfernung wird vor allem bei kleinen Kindern mit Atemproblemen vorgenommen. Die vollständige Entfernung der Gaumenmandeln ist dagegen bislang Standard zur Behandlung der wiederholten akuten Mandelentzündung (rezidivierende akute Tonsillitis).

Bislang konnte noch nicht geklärt werden, ob bei Patientinnen und Patienten mit immer wiederkehrenden akuter Mandelentzündungen, bei der der behandelnde Arzt, einen chirurgischen Eingriff empfehlen würde, eine Tonsillotomie gegenüber einer Tonsillektomie nicht unterlegen ist. Mit der Durchführung dieser Studie soll diese Fragestellung geklärt werden.

Kann ich entscheiden, welches Verfahren bei mir zur Anwendung kommt?

Einschluss in eine bestimmte operativen Gruppe

Im Rahmen dieser Studie wird die Tonsillotomie mit der Tonsillektomie verglichen, um Wirkungen und Nebenwirkungen der Verfahren besser beurteilen zu können. Beide Verfahren sind für die Therapie bei wiederkehrenden akuten Mandelentzündungen etabliert und zugelassen. Deshalb werden alle Patienten, die an der Studie teilnehmen, in zwei Gruppen eingeteilt. Die eine Gruppe erhält eine Tonsillotomie, die andere Gruppe eine Tonsillektomie.

Zu welcher Gruppe Sie im Falle Ihrer Teilnahme gehören, wird nach Zufall entschieden, vergleichbar mit dem Werfen einer Münze. Die Wahrscheinlichkeit, dass Sie eine Tonsillotomie erhalten, beträgt 50%. Der Arzt weiß aber in welcher Gruppe Sie sind.

Zielsetzung der Studie

Ziel der Studie

Das Hauptziel der Studie ist es, zu zeigen, dass die Tonsillotomie nicht weniger gut hilft als die Tonsillektomie.

Weitere Ziele

Weitere Ziele sind u.a.:

- Die systematische Erfassung von Nebenwirkungen und Anzahl der Tage an denen Halsschmerzen nach der Operation auftreten.

Wie ist der Ablauf der Studie und was muss ich bei einer Teilnahme wissen?

Voruntersuchung

Voruntersuchung

Wenn Sie bei der Studie teilnehmen, wird zuerst die Vorgeschichte Ihrer Krankheit abgefragt und Sie werden umfassend ärztlich untersucht. Dazu gehören Fragen zu Ihrer Krankheitsgeschichte und auch medizinische Untersuchungen (bspw. Gewicht, Größe). Ob Sie an der klinischen Studie teilnehmen können, hängt von den Ergebnissen dieser Voruntersuchung ab.

Ablauf der Studie und Untersuchungen im Laufe der Studie

Ablauf der Studie und Untersuchungen im Laufe der Studie

Wenn Sie der Studienteilnahme zugestimmt haben und die Eingangsuntersuchungen durchgeführt wurden, erfolgt die zufällige (= randomisierte) Zuteilung in eine der beiden Gruppen (=Behandlungsarme), wie auf der vorherigen Seite beschrieben).

• Bei der Operation werden Ihnen Ihre Gaumenmandeln teilweise oder vollständig entfernt.

• Über einen Zeitraum von 24 Monaten werden wir Ihnen wöchentlich Fragen stellen, die,

- Die Häufigkeit und Schwere Ihrer Halsschmerzen betreffen.
- Fragen zur Lebensqualität beinhalten.

Hierzu werden wir Ihnen die Möglichkeit geben, die Fragen mithilfe einer App, über eine Webseite oder eines Tagebuchs zu beantworten.

Zusätzlich werden wir Sie in regelmäßigen Abständen (und zwar alle sechs Monate über einen Zeitraum von zwei Jahren) telefonisch kontaktieren, um Ihnen Fragen zu Ihrem Wohlbefinden zu stellen. Bei eventuellen Rückfragen möchte Sie Ihr Studienzentrum (Prüfzentrum) auch gerne zwischendurch einmal kontaktieren dürfen.

Dauer der Studie

Fehler! Verweisquelle konnte nicht gefunden werden.

Die Operation und die danach folgende Behandlung dauert nur wenige Tage. Nach der Operation wollen wir jedoch über einen Zeitraum von zwei Jahren wissen, ob Sie noch weiterhin regelmäßig Halsschmerzen haben und falls ja wie stark diese Halsschmerzen sind.

Zeitlicher Ablauf der Studie

Fehler! Verweisquelle konnte nicht gefunden werden.

Der zeitliche Ablauf der Studie ist wie folgt vorgesehen:

Bezeichnung/Monat	Voruntersuchungen	Operation[a]	wöchentliche Patientendatenerhebungen (bis Monat 24)[b]	Nachuntersuchungen Monat[b] 6, 12, 18, 24
Ein-Ausschlusskriterien	X			
Patientendaten	X			
Einwilligungserklärung	X			
Randomisierung	X			
OP Daten		X		
Blutungen / UEs*		X	X	X
Anzahl Tage Halsschmerzen / NRS	X		X	X
STAR (Wenn Halschmerzen vorliegen)**			X	X
TAHSI**				X
TOI**				X
SF-12**				X

[a] = am Zentrum
[b] = Datenerhebung durch Patient [ggf. Weiterleitung Daten (bspw. Tagebuch)]
* Erfassung bei Anfall (bspw. Blutungen am behandelnden Zentrum)
** Bei Weiterleitung der Daten: Arbeitsanfall am Zentrum und zentrale Datenerfassung

Was müssen Sie bei einer Studienteilnahme beachten?

Was müssen Sie bei einer Studienteilnahme beachten?Fehler! Verweisquelle konnte nicht gefunden werden.

- Da Sie nach der Operation nicht mehr in der Klinik sein werden und in der Regel Ihren HNO Arzt nicht regelmäßig besuchen werden, ist es wichtig, dass Sie die Fragen nach Ihren Hallschmerzen wöchentlich beantworten.
- Wenn Ihrem in dieser Patienteninformation genannten behandelnden Arzt auffällt, dass Sie die Fragen nicht regelmäßig beantworten, kann es sein, dass der Arzt bei Ihnen nachfragen wird und Sie bitten, die Fragen, regelmäßig zu erfassen.

- Teilen Sie dem Studienpersonal alle Erkrankungen und Verletzungen, einschließlich eine Verschlechterung Ihres Gesundheitszustands, die während der Studie bei Ihnen auftreten, mit.

Was bringt es Ihnen, wenn Sie an der Studie teilnehme?

Was bringt Ihnen die Studienteil-nahme? Was ***bringt es Ihnen, wenn Sie an der Studie teil-nehme?***

Wenn Sie operiert werden, werden Sie längerfristig wahrscheinlich weniger Halsschmerzen haben. Wenn die Gaumenmandeln entfernt sind, können sich diese nicht mehr entzünden. Das Gewebe um die Mandeln, kann sich jedoch entzünden. Der Arzt würde Ihnen jedoch – unabhängig von der Studienteilnahme – empfehlen, dass Sie operiert werden. Hier können Sie auch eine Zweitmeinung eines anderen Arztes einholen.

Welche Risiken bestehen?

Risiken

Jede Operation, so auch eine Mandeloperation, ist mit bestimmten Risiken versehen. Dies hat nichts mit einer Studienteilnahme zu tun. Diese Risiken bestehen auch, wenn eine Mandeloperation außerhalb einer Operation vorgenommen wird.
Durch die Studienteilnahme ergibt sich also kein zusätzliches Risiko.

Bei einer teilweisen Entfernung der Gaumenmandeln (Tonsillotomie) können Nachblutungen, Blutungen, Sprachklangänderung (z.B. offenes Näseln), Schluckbeschwerden, Zahn-, Zungen-, Schleimhautschädigung und Überschlucken in die Nase als Risiken auftreten.

Bei einer kompletten Entfernung der Gaumenmandeln (Tonsillektomie) können Nachblutungen (auch bis zu 14 Tage nach Operation) mit möglicherweise letalem Ausgang, Blutungen, Sprachklangänderung (z.B. offenes Näseln), Schluckbeschwerden, Zahn-, Zungen-, Schleimhautschädigung und Überschlucken in die Nase als Risiken auftreten.

Bezüglich der Operation und den damit verbundenen speziellen Risiken werden Sie separat informiert und aufgeklärt.

Behandlungsmöglichkeiten außerhalb der Studie

Behandlungs-möglichkeiten außerhalb der Studie

Wenn Sie nicht an der Studie teilnehmen möchten, stehen zur Behandlung Ihrer Erkrankung auch die folgenden Möglichkeiten zur Verfügung: Gabe von Medikamenten (Antibiotika) zur Bekämpfung es Infekts. Da diese Behandlung bislang bei früheren Entzündungen zum Tragen kam, ohne dass eine dauerhafte Besserung eingetreten ist, würde wir Ihnen – entsprechend den aktuellen medizinischen Leitlinien – eine Operation empfehlen.

Wer darf bei dieser Studie nicht teilnehmen?

Sie dürfen nicht teilnehmen, wenn Sie gleichzeitig an anderen Studien teilnehmen oder vor kurzem teilgenommen haben.

Schwangere und stillende Frauen und Mädchen dürfen nicht teilnehmen

Schwangere und stillende Frauen dürfen an der klinischen Studie ebenfalls nicht teilnehmen.

Was kostet mich die Studie? Erhalte ich Geld zurück?

Durch Ihre Teilnahme an dieser Studie entstehen für Sie **keine** zusätzlichen Kosten. Fahrtkosten zum Studienzentrum werden nicht erstattet.

Versicherungsschutz während der Studie

Bin ich während der klinischen Studie versichert?

Sie sind während der Studie gegen eventuelle Gesundheitsschäden durch Ihre Studienteilnahme versichert. Das betrifft auch die Frage, was geschehen muss, wenn Sie den Verdacht haben, dass die Studie bei Ihnen einen Gesundheitsschaden verursacht haben könnte.

**Name der Versicherung:**	HDI Global SE
**Anschrift der Versicherung:**	Am Schönenlamp 45<br>40559 Düsseldorf
**Versicherungsnummer:**	65964770103017
**Telefon:**	0211 7482-176

Mitteilungspflicht an den Versicherer

Wenn Sie vermuten, dass durch die Teilnahme an der klinischen Studie Ihre Gesundheit geschädigt oder bestehende Leiden verstärkt wurden, müssen Sie dies unverzüglich dem Versicherer direkt anzeigen, gegebenenfalls mit Unterstützung durch Ihren behandelnden Studienarzt (Prüfarzt), um Ihren Versicherungsschutz nicht zu gefährden. Sofern Ihr Prüfarzt Sie dabei unterstützt, erhalten Sie eine Kopie der Meldung. Sofern Sie Ihre Anzeige direkt an den Versicherer richten, informieren Sie bitte zusätzlich Ihren Prüfarzt.

Mitteilung neuer Erkenntnisse während der klinischen Studie

Sie werden über neue Erkenntnisse, die in Bezug auf die Operationsmethode bekannt werden und die für Ihre Bereitschaft zur weiteren Teilnahme wesentlich sein können, informiert. Auf dieser Basis können Sie dann Ihre Entschei-

dung zur weiteren Teilnahme an dieser klinischen Studie überdenken.

Öffentliche Registrierung der Studie und Veröffentlichungen

Wo sind Informationen über diese Studie öffentlich verfügbar?

Studienregister

Diese Studie ist in einem öffentlich zugänglichen Register eingetragen. Dieses Register heißt Deutsches Register Klinischer Studien. Die Studie ist mit der Nummer DRKS00020823 dort registriert und ist unter der folgenden Webadresse verfügbar:

https://www.drks.de/drks_web/

In diesem öffentlichen Register finden Sie allgemeine Informationen über die Studie. Diese Website enthält aber keine Informationen, die zur Identifikation Ihrer Person führen.

Veröffentlichungen und Publikationen

Die Ergebnisse dieser Studie sollen veröffentlicht werden, z.B. in wissenschaftlichen Zeitschriften und auf Konferenzen. Diese Veröffentlichungen werden keinerlei persönliche Daten enthalten, die Rückschlüsse auf Ihre Person ermöglichen. Zudem ist es möglich, dass die Forschungsergebnisse kommerziell genutzt, z.B. patentiert werden. An einem möglichen kommerziellen Nutzen werden Sie nicht beteiligt.

Beendigung der klinischen Studie

Beendigung der klinischen Studie

Sie können jederzeit, auch ohne Angabe von Gründen, Ihre Teilnahme an der Studie beenden, ohne dass Ihnen dadurch irgendwelche Nachteile bei Ihrer medizinischen Behandlung entstehen.

Unter gewissen Umständen ist es aber auch möglich, dass der Prüfarzt oder der Sponsor entscheidet, Ihre Teilnahme an der klinischen Studie vorzeitig zu beenden, ohne dass Sie auf die Entscheidung Einfluss haben. Die Gründe hierfür können z. B. sein:

- Ihre weitere Teilnahme an der klinischen Studie ist ärztlich nicht mehr vertretbar;
- die gesamte klinische Studie wird abgebrochen.

Zuständige Ethikkommissionen können die Studie ebenfalls jederzeit beenden.

Sofern Sie sich dazu entschließen, vorzeitig aus der klinischen Studie auszuscheiden oder Ihre Teilnahme aus einem anderen der genannten Gründe vorzeitig beendet wird, ist es für Ihre eigene Sicherheit wichtig, dass Sie sich

einer empfohlenen abschließenden Kontrolluntersuchung unterziehen. Der Prüfarzt wird mit Ihnen besprechen, wie und wo Ihre weitere Behandlung stattfindet.

Datenschutz

Pseudonymisierung bedeutet verschlüsseltes Speichern von persönlichen Daten

Während der klinischen Studie werden medizinische Befunde und persönliche Informationen von Ihnen erhoben und in dem Prüfzentrum, in Ihrer persönlichen Akte niedergeschrieben oder elektronisch gespeichert. Die für die klinische Studie wichtigen Daten werden zusätzlich in pseudonymisierter Form gespeichert, ausgewertet und gegebenenfalls weitergegeben.

Pseudonymisiert bedeutet, dass keine Angaben von Namen oder Initialen verwendet werden, sondern nur ein Nummern- und/oder Buchstabencode.

Die Daten sind gegen unbefugten Zugriff gesichert. Eine Entschlüsselung erfolgt nur unter den vom Gesetz vorgeschriebenen Voraussetzungen.

Einzelheiten, insbesondere zur Möglichkeit eines Widerrufs, entnehmen Sie bitte der Einwilligungserklärung, die im Anschluss an diese Patienteninformation abgedruckt ist.

Für weitere Fragen

Beratungsgespräche an dem Prüfzentrum

Sie können bei jeder Gelegenheit in Ihrem behandelndem Studienzentrum (Prüfzentrum) Fragen stellen. Die Adresse finden Sie auf der Seite 1 dieser Patienteninformation.

Sie erhalten eine Kopie dieser Patienteninformation und der unterschriebenen Einverständniserklärung sowie der Versicherungsbedingungen.

Unabhängig von Ihrer Entscheidung über eine Teilnahme an der Studie, wünschen wir Ihnen für Ihren weiteren Behandlungsverlauf alles Gute.

Falls Sie keine weiteren Fragen haben und sich zur Teilnahme an dem wissenschaftlichen Projekt entschieden haben, unterzeichnen Sie bitte die beiliegende Einverständniserklärung.

Persönliche Notizen

Einverständniserklärung

Toto: Tonsillektomie versus Tonsillotomie bei Kindern und Erwachsenen mit rezidivierender akuter Tonsillitis: Eine kontrollierte, randomisierte Nichtunterlegenheits-Studie

**Prüfzentrum** *(Stempel):*	**Prüfarzt:**	
	Name:	______________________
	Telefon:	______________________

Name des Patienten in Druckbuchstaben

/ /

geboren am

/

Zentrums-Nr. / Patienten-Nr.

Ich bin in einem persönlichen Gespräch durch den Prüfarzt

Name der Ärztin / des Arztes

ausführlich und verständlich über den operativen Eingriff sowie über Wesen, Bedeutung, Risiken und Tragweite der klinischen Studie aufgeklärt worden. Ich habe darüber hinaus den Text der Patienteninformation sowie die hier nachfolgend abgedruckte Datenschutzerklärung gelesen und verstanden. Ich hatte die Gelegenheit, mit dem Prüfarzt über die Durchführung der klinischen Studie zu sprechen. Alle meine Fragen wurden zufriedenstellend beantwortet.

Möglichkeit zur Dokumentation zusätzlicher Fragen seitens des Patienten oder sonstiger Aspekte des Aufklärungsgesprächs:

____

____

____

____

Ich hatte ausreichend Zeit, mich zu entscheiden.

Mir ist bekannt, dass ich jederzeit und ohne Angabe von Gründen meine Einwilligung zur Teilnahme an der Studie zurückziehen kann (mündlich oder schriftlich), ohne dass mir daraus Nachteile für meine medizinische Behandlung entstehen.

Datenschutz

Mir ist bekannt, dass bei dieser klinischen Studie personenbezogene Daten, insbesondere medizinische Befunde über mich erhoben, in pseudonymisierter Form gespeichert und ausgewertet werden. Die personenbezogenen Daten werden nur nach entsprechender Anonymisierung an Dritte weitergegeben. Die Verwendung der Angaben über meine Gesundheit erfolgt nach gesetzlichen Bestimmungen und setzt vor der Teilnahme an der klinischen Studie folgende freiwillig abgegebene Einwilligungserklärung voraus, das heißt ohne die nachfolgende Einwilligung kann ich nicht an der klinischen Studie teilnehmen.

1. Ich erkläre mich damit einverstanden, dass im Rahmen dieser klinischen Studie personenbezogene Daten, insbesondere Angaben über meine Gesundheit, über mich erhoben und in Papierform sowie auf elektronischen Datenträgern im

   ....

   (Prüfzentrum hier eintragen)

   aufgezeichnet werden. Soweit erforderlich, dürfen die erhobenen Daten pseudonymisiert (verschlüsselt) weitergegeben werden:

   a) an den Sponsor, die HNO-Klinik Universitätsklinikum Jena oder eine von diesem beauftragte Stelle zum Zwecke der wissenschaftlichen Auswertung,

   b) an das Studienzentrum der Universitätsmedizin Göttingen zur Organisation aller für die klinische Studie relevanten Tätigkeiten,

   c) im Falle unerwünschter Ereignisse: im Auftrag des Sponsors an das Studienzentrum der Universitätsmedizin Göttingen und an die jeweils zuständige Ethik-Kommission

   d) Außerdem erkläre ich mich damit einverstanden, dass autorisierte und zur Verschwiegenheit verpflichtete Beauftragte des Sponsors in meine, beim Prüfarzt vorhandenen personenbezogenen Daten, insbesondere meine Gesundheitsdaten, Einsicht nehmen, soweit dies für die Überprüfung der ordnungsgemäßen Durchführung der Studie notwendig ist.

2. Die Einwilligung zur Erhebung und Verarbeitung meiner personenbezogenen Daten, insbesondere der Angaben über meine Gesundheit, kann von mir jederzeit widerrufen werden. Ich bin bereits darüber aufgeklärt worden, dass ich jederzeit die Teilnahme an der klinischen Studie beenden kann. Im Fall eines solchen Widerrufs meiner Einwilligung, an der Studie teilzunehmen, erkläre ich mich

   ☐ **damit einverstanden**, dass die bis zu diesem Zeitpunkt gespeicherten Daten ohne Namensnennung/Personenbezug weiterhin verwendet werden dürfen

   ☐ **nicht damit einverstanden**, dass die bis zu diesem Zeitpunkt gespeicherten Daten nicht weiterhin verwendet werden. Meine Daten müssen in diesem Fall im Rahmen der technischen Möglichkeiten gelöscht, bzw. anonymisiert werden.

3. Ich erkläre mich damit einverstanden, dass meine Daten nach Beendigung oder

Abbruch der Studie mindestens zehn Jahre aufbewahrt werden. Danach werden meine personenbezogenen Daten gelöscht, soweit nicht gesetzliche Aufbewahrungsfristen entgegenstehen.

Ich bin über folgende gesetzliche Regelung informiert: Falls ich meine Einwilligung, an der Studie teilzunehmen, widerrufe, müssen alle Stellen, die meine personenbezogenen Daten, insbesondere Gesundheitsdaten, gespeichert haben, unverzüglich prüfen, inwieweit die gespeicherten Daten noch erforderlich sind. Nicht mehr benötigte Daten sind unverzüglich zu löschen.

4. Ich bin damit einverstanden, dass ich innerhalb des Nachuntersuchungszeitraums regelmäßig (und zwar alle sechs Monate über einen Zeitraum von zwei Jahren) und eventuell zwischenzeitlich, telefonisch kontaktiert werden darf, um Fragen zu meinem Wohlbefinden zu erhalten, durch

   ………………………………………………………………………………………………

   (Prüfzentrum hier eintragen)

5. Ich bin damit einverstanden, dass mein Hausarzt / niedergelassener behandelnder Arzt

   ………………………………………………………………………………………………
   (Namen hier eingetragen)

   über meine Teilnahme an der klinischen Studie informiert wird (falls nicht gewünscht, bitte streichen).

6. Der Verantwortliche für die Datenerhebung dieser klinische Studie ist:

   Universitätsmedizin Göttingen, Studienzentrum UMG, erreichbar über:

   Von Bar Str. 2/4, 370775 Göttingen, Tel. 0551-39-60812

7. Der für diese klinische Studie verantwortliche Datenschutzbeauftragte ist:

Datenschutzbeauftragter des Universitätsklinikums Jena

Zentrum für Gesundheits-und Sicherheitsmanagement,

Beauftragte für Datenschutz des Universitätsklinikum Jena

Adresse: Bachstraße 18, 07743 Jena

Telefon: 03641 9-325 624

Fax: 03641 9-399 925

E-Mail: Datenschutzbeauftragter@med.uni-jena.de

8. Mir ist bekannt, dass ich bezogen auf die Verarbeitung der personenbezogenen Daten ein Beschwerderecht bei einer Datenschutz-Aufsichtsbehörde habe. Dies ist für Thüringen:

Thüringer Datenschutzaufsichtsbehörde

Thüringer Landesbeauftragter für den Datenschutz und die Informationsfreiheit (TLfDI)

Adresse: Postfach 900455, 99107 Erfurt

Telefon: 0361 57-311 29 00

Fax: 0361 57-311 29 04

E-Mail: poststelle@datenschutz.thueringen.de

9. Mir ist bekannt, dass ich bezogen auf die Verarbeitung der personenbezogenen Daten ein Beschwerderecht bei der Bundesdatenschutzbeauftragte habe:

Bundesdatenschutzbeauftragte:

Die Bundesbeauftragte für den Datenschutz und die Informationsfreiheit

Adresse: Husarenstr. 30, 53117 Bonn

Telefon: 0228 997799-0

Fax: 0228 997799-550

E-Mail: poststelle@bfdi.bund.de

10. Mir ist bekannt, dass ich ein Recht auf Auskunft über die verarbeiteten personenbezogenen Daten habe. Dieses Auskunftsrecht besteht gegenüber dem unter Punkt 7 genannten Verantwortlichen.
11. Mir ist bekannt, dass ich ein Recht auf unverzügliche Berichtigung (eingeschränkt) Übertragung und Löschung meiner personenbezogenen Daten habe.

PATIENT

Mit meiner Unterschrift erkläre ich mit bereit, an der oben genannten klinischen Studie freiwillig teilzunehmen.

Eine Kopie der Patienteninformation und -Einwilligung sowie die Versicherungsbedingungen habe ich erhalten. Ein Exemplar verbleibt im Prüfzentrum.

Name des Patienten in Druckbuchstaben
(eigenhändig vom Patienten einzutragen)

/ /

Datum
(eigenhändig vom Patienten einzutragen)

Unterschrift des Patienten

ARZT

Ich habe das Aufklärungsgespräch geführt und die Einwilligung des Patienten eingeholt.

Name der Prüfärztin / des Prüfarztes in Druckbuchstaben

/ /

Datum

Unterschrift der Prüfärztin / des Prüfarztes in Druckbuchstaben
